# Dendritic Cells: Origin, Classification, Development, Biological Functions, and Therapeutic Potential

**DOI:** 10.1002/mco2.70455

**Published:** 2025-11-05

**Authors:** Fangfang Jin, Lingxiang Xie, Hongqi Zhang, Xiang Fan, Jiaxing Tian, Wei Liu, Yang Xiao, Xinrong Fan

**Affiliations:** ^1^ Experimental Research Center, China Academy of Chinese Medical Sciences Beijing China; ^2^ National Clinical Research Center for Metabolic Diseases, Key Laboratory of Diabetes Immunology, Ministry of Education, and Department of Metabolism and Endocrinology, The Second Xiangya Hospital of Central South University Changsha China; ^3^ Beijing University of Chinese Medicine Beijing China; ^4^ Institute of Metabolic Diseases, Guang'anmen Hospital, China Academy of Chinese Medical Sciences Beijing China

**Keywords:** biological functions, classification, dendritic cells, development, origin, therapeutic potential

## Abstract

Dendritic cells (DCs) are professional antigen‐presenting cells (APCs) that play a central role in regulating immune responses by linking innate and adaptive immunity. In recent decades, substantial progress has been made in understanding the development, classification, and diverse functions of DCs. However, a comprehensive overview integrating recent advances in the biology and therapeutic targeting of DCs remains lacking. This review systematically summarizes the origin, developmental pathways, and subset heterogeneity of DCs, including classical type 1 and 2 DCs, plasmacytoid DCs, monocyte‐derived DCs, and Langerhans cells. Moreover, it further details the core biological functions of DCs, including antigen capture, migration, and maturation; antigen presentation; activation of adaptive immunity; induction of immune tolerance; and modulation of innate immune responses. The pathological roles of DCs in diseases such as cancer, diabetes, and infectious diseases are discussed, highlighting emerging DC‐based therapeutic strategies. Importantly, this review provides a summary of both preclinical studies and clinical trials involving DC‐targeted therapies, offering a translational perspective. This work aims to deepen the understanding of DC immunobiology and to provide a valuable foundation for the development of novel DC‐based immunotherapies.

## Graphical abstract

This review systematically summarizes dendritic cells (DCs) originating from hematopoietic stem cells, their major subsets (cDC1s, cDC2s, pDCs, moDCs, LCs) and core functions (antigen capture/presentation, adaptive immunity activation, immune tolerance induction, and regulation of innate immune cells). It further elaborates DCs’ pathological roles in cancer, diabetes, infectious diseases, and other conditions, as well as therapeutic strategies like DC vaccines, tolerogenic DCs, and combined nanotechnology/checkpoint inhibitors. Current challenges (limited antigen selection, insufficient durability of tolerance induction, low targeting efficiency, and impact of individual heterogeneity, etc.) are also highlighted to guide future DC‐based immunotherapy development.

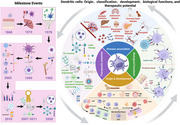

## 1 Introduction

Dendritic cells (DCs), professional antigen‐presenting cells (APCs) with unique immunological functions, play essential roles in both immune defenses and the maintenance of immune homeostasis [1]. Since their initial discovery and naming by Ralph Steinman and Zanvil Cohn in 1973, research on DCs has spanned half a century [2]. Early studies revealed their distinctive dendritic morphology and potent capacity to activate naïve T cells [3]. This discovery challenged the contemporary understanding of immune activation, laid the groundwork for elucidating the mechanisms initiating adaptive immunity, and ultimately earned Steinman the 2011 Nobel Prize in Physiology or Medicine. Initial research focused primarily on DC morphology, isolation methods, and robust stimulatory activity in mixed lymphocyte reactions, progressively establishing their central role in the immune system [4, 5].

Advances in technologies such as flow cytometry, gene knockout models, and, particularly, single‐cell omics have led to rapid advancements in DC research. Recent studies have elucidated the complex ontogeny and developmental pathways of DCs, revealing their origin from distinct branches of bone marrow hematopoietic stem cells (HSCs) under precise transcriptional regulation [6, 7, 8]. Based on their developmental origin, phenotype, tissue distribution, and functional specialization, DCs are classified into several subsets: classical type 1 and 2 DCs (cDC1s and cDC2s), plasmacytoid DCs (pDCs), monocyte‐derived DCs (moDCs), and Langerhans cells (LCs) [9, 10, 11]. Although each subset performs specialized functions, they collectively act as sentinels of the immune system that are adept at capturing, processing, and presenting antigens [9, 12, 13]. Upon sensing danger signals, they undergo maturation and migrate to secondary lymphoid organs, where they activate antigen‐specific T cells and B cells, thereby initiating and shaping adaptive immune responses [9, 12, 13]. Furthermore, DCs are indispensable for maintaining immune tolerance and regulating innate immune cells such as natural killer (NK) cells and natural killer T (NKT) cells [14, 15]. Accordingly, the dysregulation of DC function is associated with the pathogenesis of various major diseases, including cancer, autoimmune disorders (e.g., type 1 diabetes mellitus [T1DM]), infectious diseases, and transplant rejection [16, 17, 18, 19].

Despite considerable progress, several challenges remain in the field: the regulatory networks governing the development and functions of different DC subsets remain incompletely elucidated; the functional states and interactions of DC subpopulations within complex disease microenvironments require further exploration; and strategies to efficiently translate basic research into clinical applications need optimization. In recent years, DC‐based therapeutic strategies, particularly cancer vaccines, have shown promising potential in the field of tumor immunotherapy. Exploratory applications in other diseases, such as diabetes, anti‐infection therapies, and transplant tolerance induction, are also becoming increasingly active [20, 21, 22, 23, 24, 25]. However, a gap remains in the literature regarding comprehensive reviews that integrate basic DC biology (origin, classification, development, and function) with their therapeutic potential across diverse disease contexts.

This review aims to provide an integrated overview of DC biology and therapeutic potential, charting the progression from fundamental mechanistic insights to clinical translation. Specifically, this review will (i) describe the origin and developmental processes of DCs; (ii) summarize the key characteristics of different DC subsets; (iii) discuss the core biological functions of DCs; and (iv) examine the mechanisms and therapeutic potential of DCs in various diseases. Additionally, this review will synthesize key findings from both preclinical and clinical studies involving DC‐based interventions, providing a critical appraisal of current strategies and future directions in DC immunotherapy.

## 2 Origins and Development of DCs

**FIGURE 1:**
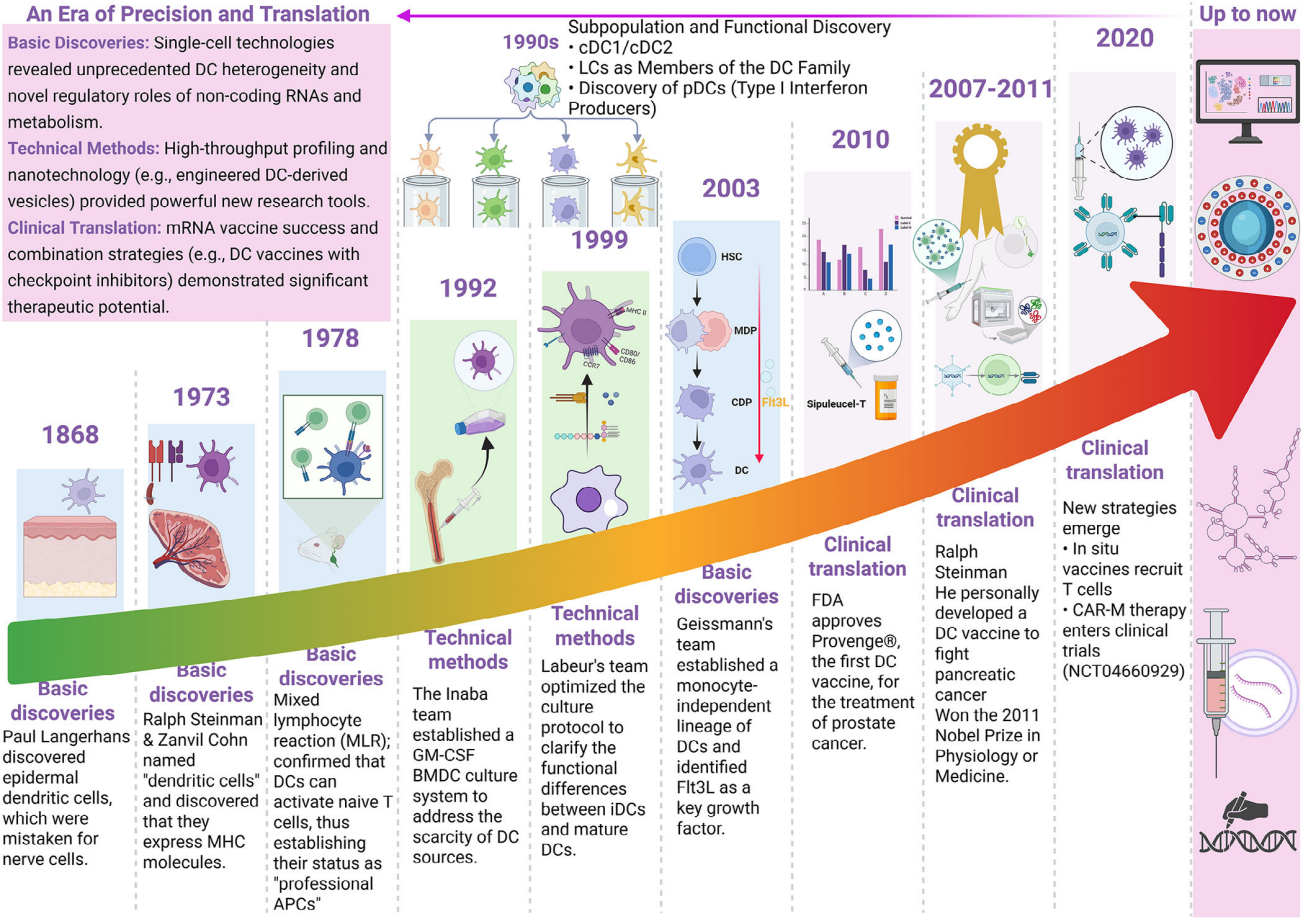
Milestones in the history of dendritic cell research. This timeline delineates the seminal events that have defined the understanding and application of dendritic cells (DCs). It begins with their initial identification and establishment as professional antigen‐presenting cells (APCs), followed by critical methodological advances that enabled their in vitro study. The journey culminates in clinical breakthroughs, from the first approved DC vaccine to modern strategies like in situ vaccination and CAR‐M therapy, and enters a contemporary era of precision medicine driven by single‐cell technologies and novel regulatory insights.

DCs originate from HSCs and undergo a series of lineage‐specific differentiation steps to generate functionally distinct subsets with diverse phenotypes. This process is tightly regulated by key transcription factors (e.g., interferon regulatory factor 8 [IRF8], PU.1, inhibitor of DNA binding 2 [ID2], and Notch) and cytokines (such as Fms‐like tyrosine kinase 3 ligand [Flt3L] and granulocyte‒macrophage colony‐stimulating factor [GM‐CSF]). Understanding DC developmental pathways and their regulatory mechanisms is essential for deciphering the heterogeneity and functional specificity of DC subsets. This section systematically revisits major milestones in DC research (Figure 1), delves into their developmental trajectories from HSCs to functionally distinct subsets, and elucidates the key regulatory networks governing these processes.

### 2.1 History of DC Research and Milestone Events

The history of DC research constitutes a cornerstone of modern immunology. In 1868, the German medical student Paul Langerhans first observed cells with a dendritic morphology in the epidermis [26]. However, limited by contemporary knowledge, he mistakenly classified them as neuronal cells [26]. In 1973, Canadian scientists Ralph Steinman and Zanvil Cohn identified a unique population of stellate immune cells in the mouse spleen and peripheral lymph nodes and named them “dendritic cells” based on their extensive cytoplasmic projections [27, 28]. They also demonstrated that DCs expressed high levels of major histocompatibility complex (MHC) molecules, suggesting a potent antigen‐presenting capacity. In 1978, mixed lymphocyte reaction experiments confirmed that DCs activate naïve T cells via MHC–antigen peptide complexes, establishing DCs as professional APCs [29].

By the 1990s, DC research had shifted toward subset differentiation and functional specialization. Investigators discovered that splenic DCs can be subdivided into CD8α⁺ and CD11b⁺ populations with distinct immunological effects [30]. Concurrently, epidermal LCs were identified as members of the DC family, whose migratory capacity and immune‐activating properties expand the concept of “sentinels” in immunity [31]. Another milestone was the discovery of pDCs: these cells secrete large amounts of type I interferon (IFN‐I) upon viral stimulation and can differentiate into immunostimulatory DCs [32]. This period also featured key methodological advances. In 1992, Inaba's team established a GM‐CSF‐induced bone marrow‐derived DC (BMDC) culture system, overcoming the challenge of DC scarcity in vivo [33]; in 1999, Labeur et al. [34] optimized culture protocols using IL‐4 together with a CD40 ligand (CD40L) or lipopolysaccharide (LPS), enabling the functional modulation of DCs from an “immature” state (high antigen capture) to a “mature” state (strong T‐cell activation).

The theory of DC ontogeny underwent a significant revision in the 21st century. The conventional view that DCs originate from monocytes was challenged in 2003 by the team of Frédéric Geissmann. Through genetic lineage tracing and bone marrow chimera experiments, they showed that under steady‐state conditions, tissue‐resident DCs and LCs are derived from specific hematopoietic progenitors independent of monocytes (e.g., macrophage–DC progenitors [MDPs] and common DC progenitors [CDPs] in mice) and that the Flt3L signaling pathway is a central regulator of DC development [35]. This discovery established DCs as an independent hematopoietic lineage. Clinical applications have also yielded landmark progress. In 2010, the United States Food and Drug Administration approved the first DC‐based vaccine, Provenge (sipuleucel‐T), for prostate cancer, extending the median survival by 4.1 months [36]. An even more symbolic milestone was Steinman's own experience: following his diagnosis with advanced pancreatic cancer in 2007, he designed three autologous DC vaccine regimens (peptide pulsing, RNA transfection, and T‐cell coadministration), extending his survival to 4.5 years. He was awarded the 2011 Nobel Prize in Physiology or Medicine three days after his death, underscoring the transformative impact of DC research. In recent years, innovative strategies have been developed, such as the CCL21–DC in situ tumor vaccine developed at the University of California to recruit T cells into lung tumors [37] and the first clinical trial for chimeric antigen receptor macrophage therapy in 2020, opening new avenues for DC‐derived cell therapies [38]. Since 2020, DC research has achieved multiple landmark breakthroughs. In fundamental discoveries, single‐cell sequencing and related technologies have enabled a more refined understanding of DC heterogeneity and differentiation pathways, leading to the identification of novel DC subsets and revealing the critical roles of noncoding RNAs, metabolic reprogramming, and sex hormones in regulating DC function [39, 40, 41]. Technologically, high‐throughput single‐cell approaches and multiplex imaging have revolutionized DC research paradigms, while novel computational models [42], genetically engineered DC vaccines [43], and nanotechnology such as DC‐derived nanovesicles [44] have provided powerful tools for DC investigation and application. In clinical translation, the success of mRNA COVID‐19 vaccines stands as a major milestone [45]; their mechanism of efficiently activating DCs has provided valuable insights for cancer vaccine development. Meanwhile, combination therapies of DC vaccines with immune checkpoint inhibitors [46], neoantigen‐based personalized vaccines [47], and in vivo DC reprogramming strategies have demonstrated substantial potential in clinical trials [48]. Additionally, tolerogenic DCs (tolDCs) have entered early‐stage clinical exploration for autoimmune disease treatment [23].

### 2.2 Origins and Processes of DC Development

**FIGURE 2:**
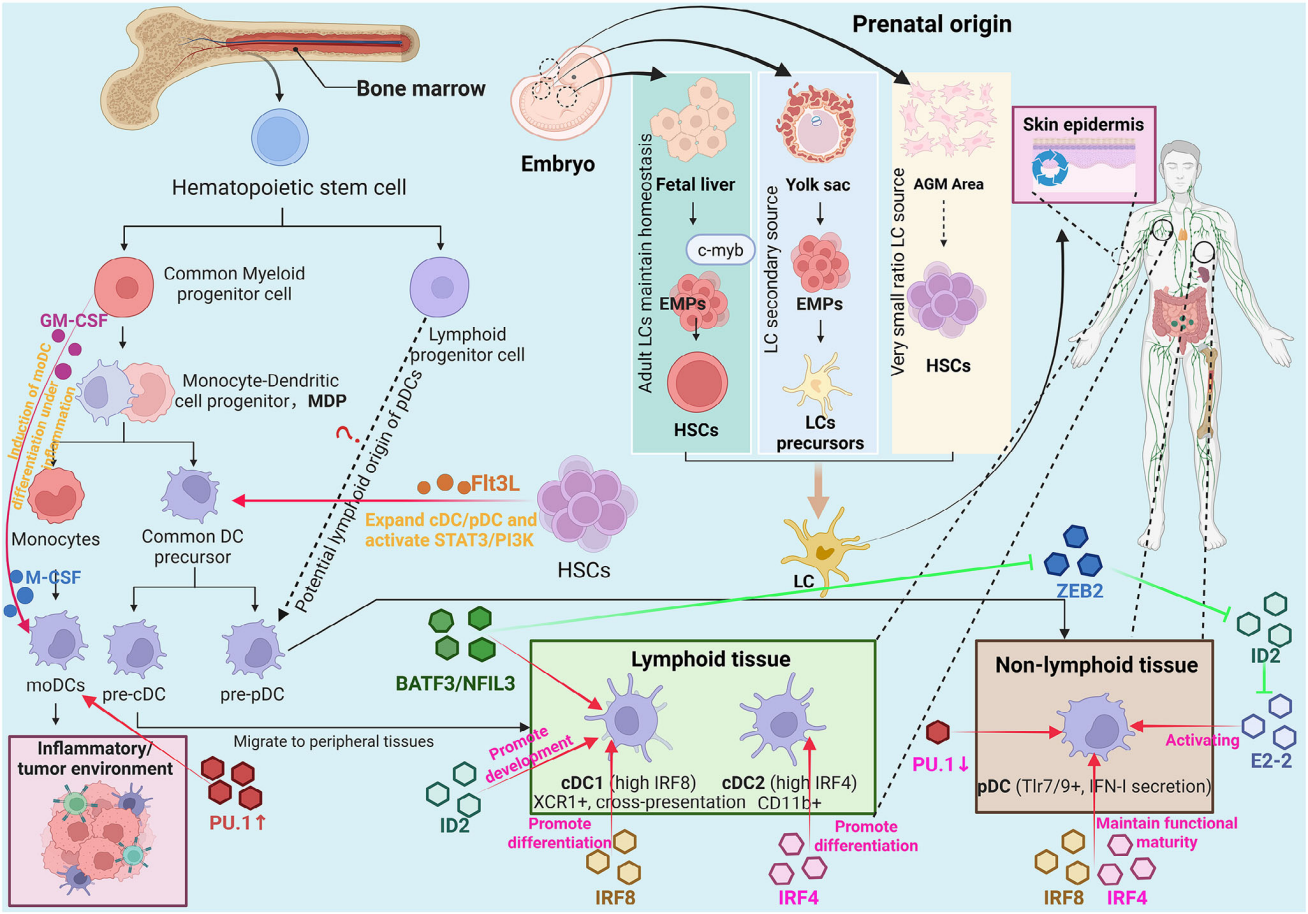
The origins, developmental pathways, and regulatory networks of dendritic cells. Dendritic cells (DCs) originate from hematopoietic stem cells (HSCs) in the bone marrow. HSCs differentiate into myeloid precursors (MPs) and lymphoid precursors (LPs), giving rise to various DC subsets through distinct pathways. In the myeloid pathway, MPs give rise to macrophage–DC precursors (MDPs), which subsequently generate common DC precursors (CDPs). CDPs further differentiate into preplasmacytoid DCs (pre‐pDCs) and preclassical DCs (pre‐cDCs). Pre‐cDCs migrate to lymphoid and nonlymphoid tissues to develop into two major subsets: cDC1s and cDC2s. pDCs are suggested to have dual origins from both myeloid cells and LPs. In contrast, monocyte‐derived DCs (moDCs) are generated from monocytes only under inflammatory conditions. Langerhans cells (LCs) primarily originate from embryonic yolk sac‐derived erythromyeloid progenitors (EMPs) and fetal liver monocytes, with a minor contribution from HSCs, and are maintained in adults through local self‐renewal. The development of DCs is tightly regulated by key cytokines (e.g., Flt3L, GM‐CSF, and M‐CSF) and a complex transcriptional network (e.g., IRF8, IRF4, ID2, E2‐2, and BATF3), which determines the commitment and differentiation of specific DC subsets. Red and green arrows: molecular regulatory pathways (red indicates facilitation, green indicates inhibition); black arrows indicate origins and developmental pathways;? represents controversial findings. *Abbreviations*: Flt3L, Fms‐related tyrosine kinase 3 ligand; GM‐CSF, granulocyte‒macrophage colony‐stimulating factor; M‐CSF, macrophage colony‐stimulating factor; IRF8, interferon regulatory factor 8; IRF4, interferon regulatory factor 4; ID2, DNA‐binding protein inhibitor ID2; E2‐2, transcription factor E2‐2; BATF3, basic leucine zipper ATF‐like transcription factor 3.

DCs originate from resident hematopoietic progenitors in the bone marrow. HSCs differentiate into DC‐committed progenitors, including myeloid precursors (MPs) and lymphoid precursors (LPs), which give rise to various DC subsets through myeloid or lymphoid developmental pathways (Figure 2) [49]. In the myeloid pathway, MPs differentiate into macrophages, monocytes, and MDPs, which subsequently generate monocytes and CDPs. CDPs produce pre‐pDCs and pre‐cDCs [49]. Pre‐pDCs develop into pDCs, while pre‐cDCs migrate to lymphoid and nonlymphoid tissues to differentiate into the cDC1 and cDC2 subsets [49]. In contrast to the exclusively myeloid origin of cDCs, pDCs may exhibit a dual developmental origin from both myeloid and lymphoid progenitors. Several studies suggest that LPs can also differentiate into pDCs [49, 50, 51, 52]. For instance, Rag1 expression during pDC development supports a lymphoid origin [49, 52], and lineage tracing driven by the Il7ra or Csf1r promoter further supports this possibility [50, 51]. However, pDCs share many features that are more closely aligned with cDCs than with lymphocytes [53]. First, both pDCs and cDCs develop through a Flt3L‐dependent pathway [54, 55]. While Flt3L promotes the generation of both pDCs and cDCs, it is not required for the development of B cells or innate lymphoid cells (ILCs) (Figure 2) [56, 57]. Second, pDC development is independent of IL‐2Rγ, whereas B cells and ILCs rely on IL‐2Rγ–Janus kinase (JAK)/signal transducer and activator of transcription (STAT) signaling [58, 59]. Furthermore, pDC development remains unaffected by the depletion of lymphoid progenitors, and their lifespan more closely resembles that of cDCs [59]. Thus, the developmental origin of pDCs requires further investigation. moDCs are nonhomeostatic cells generated from monocytes specifically under inflammatory conditions, such as infection or within the tumor microenvironment (TME) [60]. LCs were initially thought to originate from the bone marrow [61]. However, recent studies have revealed a more complex developmental pathway involving primitive erythromyeloid progenitors (EMPs) in the yolk sac and fetal liver monocytes (Figure 2). Studies have shown that the first wave of primitive myeloid cells, which is derived from yolk sac EMPs, gives rise to yolk sac macrophages [62, 63]. These cells migrate to the epidermis around embryonic Day 7.5, proliferate, and generate LCs, which persist into adulthood [62, 63]. Another study revealed a distinct late c‐myb⁺ EMP subset that produces fetal liver monocytes, which serve as key precursors of LCs [64]. Additionally, a small fraction of LCs originates from HSCs in the aorta–gonad–mesonephros region. Three weeks after birth, the adult LC network is established and maintained through local self‐renewal, with no contribution from HSC‐derived cells [65]. Following severe inflammation, the LC network can be replenished by BMDCs [62].

### 2.3 Cytokine Regulation of DC Development

The development of DCs is regulated by multiple cytokines, including Flt3, GM‐CSF, and M‐CSF (Figure 2). Flt3 is a surface protein that is highly expressed on HSCs, and together with its ligand Flt3L, it drives the proliferation, differentiation, and development of cDCs both in vivo and in bone marrow cultures [66, 67]. Flt3L markedly expands splenic DC subsets coexpressing MHC‐II, CD11c, DEC205, and CD8 [68]. Research by Schlaweck et al. [69] demonstrated that the Flt3L inhibitor midostaurin reduces the frequency of mature DCs differentiated from mouse bone marrow cells, significantly impairing DC differentiation, which highlights the essential role of Flt3L in DC development. Moreover, a recent single‐cell analysis revealed that Flt3L promotes DC development by maintaining hematopoietic stem and progenitor cells in a proliferative “early progenitor” state, leading to the selective expansion of transitional progenitor stages biased toward cDC1 lineage commitment [70]. Flt3 signals through the mitogen‐activated protein kinase (MAPK), phosphatidylinositol 3‐kinase (PI3K), and STAT3 pathways, with the PI3K and STAT3 pathways being particularly critical for DC development [67]. GM‐CSF is a cytokine that stimulates myeloid colony formation from bone marrow precursors. One study reported that after bone marrow cells were cultured with GM‐CSF for 7 days, 18% of the total adherent cells were GM‐CSF‐grown BMDCs [71]. Ryu et al. [72] injected mice with various hematopoietic cytokines and found that only GM‐CSF generated distinct subsets of XCR1^−^33D1^−^ DCs, which constituted the majority of splenic DCs after treatment. GM‐CSF‐deficient mice exhibit a significant reduction in DC numbers, highlighting its vital role in DC development [73]. Similarly, GM‐CSF is indispensable for the generation of human DCS/LCs [74]. M‐CSF, a myelopoietic cytokine that is released during infection and inflammation, directly induces the expression of the myeloid master regulator PU.1 and directs myeloid fate changes in mouse HSCs [75]. Yao and Tseng [76] first reported that in serum‐free culture, the addition of low‐dose interleukin (IL)‐6 and M‐CSF synergized with GM‐CSF and IL‐4 to yield greater numbers of functionally superior DCs than GM‐CSF and IL‐4 alone, suggesting that M‐CSF contributes to DC development. Moreover, a recent study established a culture system in which human monocytes differentiated into both DCs and macrophages upon stimulation with M‐CSF, IL‐4, and tumor necrosis factor‐alpha (TNF‐α) [77].

### 2.4 Transcriptional Regulation of DC Development

The development of DCs is governed by a complex and highly coordinated network of transcription factors, including IRF family members, the E‐protein/ID inhibitor axis, ETS family proteins, zinc finger E‐box binding homeobox 2 (ZEB2), basic leucine zipper (bZIP) transcription factor ATF‐like 3 (BATF3), and NOTCH signaling factors. Among them, IRF8 and IRF4 are central regulators. High IRF8 expression drives cDC1 differentiation (Figure 2), which is initiated in early DC progenitors under the control of the +56 kb enhancer (regulated by RUNX/CBFβ) and is maintained at the pre‐cDC1 stage via a +32 kb enhancer (Figure 2) [78, 79, 80, 81, 82]. In contrast, cDC2 development requires low levels of IRF8 but high levels of IRF4 expression, whereas pDCs depend on both IRF8 and IRF4 for functional maturation (Figure 2) [83]. Unlike cDC1s, moDC development relies on low levels of IRF8, which is also regulated by the +56 kb enhancer [79]. The E protein family member E2‐2 and its antagonist ID2 are indispensable for pDC and cDC1 differentiation, respectively. E2‐2 exists in long (E2‐2L) and short (E2‐2S) isoforms, with E2‐2L preferentially expressed in pDCs, where it maintains E2‐2S expression through pDC‐specific enhancers [84]. E2‐2S forms a complex with the myeloid translocation gene Mtg16 on chromosome 16, controlling the expression of key genes required for pDC development and function [85]. By suppressing the DNA‐binding activity of E2‐2, ID2 blocks pDC differentiation and promotes cDC1 development [80]. This antagonistic relationship is modulated by ZEB2, which suppresses ID2 expression, thereby alleviating the inhibition of E2‐2 activity and promoting pDC differentiation (Figure 2) [86]. bZIP family factors (NFIL3 and BATF3) are necessary for cDC1 development (Figure 2) [86]. NFIL3 binds to the −165 kb Zeb2 enhancer in CDPs, preventing ZEB2 expression and thus relieving the inhibition of ID2 to facilitate cDC1 differentiation [80, 87]. The ETS family members PU.1 and Spi‐B exhibit dynamic expression patterns across DC subsets. High PU.1 expression maintains cDC identity, whereas PU.1‐deficient cDCs acquire pDC‐like features, suggesting that the downregulation of PU.1 expression in progenitors is a critical permissive step for pDC differentiation [88, 89]. Furthermore, PU.1 expression is negatively regulated by BCL11A, a key regulator of pDC development [90]. Studies also indicate that MafB expression drives human monocyte differentiation toward macrophages, while high PU.1 levels promote moDC differentiation by suppressing MafB expression [91, 92]. In contrast, Spi‐B expression increases significantly from progenitors to mature pDCs [93]. Germline deletion of Spi‐B reduces pDC numbers in the bone marrow but increases them in peripheral organs [93]. Notch signaling factors (RBPJ and NOTCH2) and Krüppel‐like factors (KLF4) regulate the heterogeneous development of cDC2 subsets [86]. RBPJ is enriched in the cDC2a subset, whereas KLF4 specifically regulates the ESAM^−^ cDC2 subset, indicating that Notch signaling and KLF4 may independently regulate distinct cDC2 branches [94, 95, 96]. The RUNX family (RUNX/CBFβ and RUNX2) also participates in DC development. RUNX/CBFβ activates the +56 kb Irf8 enhancer to initiate IRF8 expression, whereas RUNX2 is essential for promoting pDC differentiation in the bone marrow [79, 97, 98].

In summary, DC development is a multilayered process regulated by diverse cytokines and transcriptional networks, with each subset following distinct differentiation pathways and functional trajectories. This developmental diversity underpins their functional specialization and context‐specific immune responses. The following section systematically discusses the classification and heterogeneity of DC subsets.

## 3 Classification of DCs

DCs are categorized into several subsets based on their developmental origin, surface marker expression, and specialized immune functions. The primary subsets include cDC1s, cDC2s, pDCs, moDCs, and LCs (Table 1). Each subset plays distinct roles in antigen presentation, cytokine secretion, and the regulation of immune responses.

**TABLE 1:** Major human dendritic cell subset characterization.

Human DC subset	Location	Transcription factors	Cytokines profile	TLR expression	Functions	Citations
cDC1s	Blood, tissue, and lymphoid organs	IRF8, NFIL3, BATF3, PU.1, ID2	IL‐12, TNF, IL‐6, IFN‐I, IFN‐III, IFN‐γ, CXCL9, CXCL10	1, 3, 6, 8, 10	cDC1s mediate antitumor immunity by cross‐presenting antigens to activate CD8⁺ T cells.	[99, 100, 101, 102, 103, 104, 105]
cDC2s	Blood, tissue, and lymphoid organs	IRF4, ID2, ZEB2, KLF4, NOTCH2	IL‐1β, IL‐8, IL‐6, IL‐12, IL‐16, IL‐23, IL‐10, TNF, TGF‐β	1, 2, 3, 4, 5, 6, 7, 8, 10	cDC2s orchestrate CD4⁺ T helper cell responses against extracellular pathogens like bacteria and fungi.	[106, 107, 108, 109, 110, 111, 112, 113]
pDCs	Blood, tissue, and lymphoid organs	IRF4, IRF7, IRF8, ZEB2, E2‐2	IFN‐I, IFN‐III, TNF, IL‐6, IL‐12 p40	7, 9, 10	pDCs play a crucial role in antiviral immunity, particularly in sensing viral infections and producing interferons.	[19, 55, 114, 115, 116, 117]
moDCs	Site of inflammation	IRF4, MAFB, KLF4, ZBKB46	IL‐1β, IL‐6, IL‐10, IL‐12, IL‐23, TNF, iNOS	1, 2, 3, 4, 7, 8	moDCs are monocyte‐derived cells that present antigen and produce inflammatory cytokines at infection sites.	[118, 119, 120, 121]
LCs	Epidermis, stratified squamous epithelia	RUNX3, ID2	IL‐15	1, 2, 3, 6, 10	LCs are epidermal immune sentinels that capture antigens, migrate to lymph nodes, and balance immune tolerance with activation.	[122, 123, 124, 125, 126, 127, 128]

Abbreviations: BATF3, basic leucine zipper ATF‐like transcription factor 3; cDC, conventional dendritic cell; cDC1s, type 1 conventional dendritic cells; cDC2s, type 2 conventional dendritic cells; CXCL, C‐X‐C motif chemokine ligand; DC, Dendritic cells; E2‐2, transcription factor E2‐2; ID2, inhibitor of DNA binding 2; IFN, interferon; IL, interleukin; iNOS, inducible nitric oxide synthase; IRF, interferon regulatory factor; KLF4, Krüppel‐like factor 4; LCs, Langerhans cells; MAFB, MAF bZIP transcription factor B; moDCs, monocyte‐derived dendritic cells; NFIL3, nuclear factor interleukin 3 regulated; NOTCH2, neurogenic locus notch homolog protein 2; pDCs, plasmacytoid dendritic cells; PU.1, transcription factor PU.1; RUNX3, runt‐related transcription factor 3; TGF‐β, transforming growth factor beta; TLR, Toll‐like receptor; TNF, tumor necrosis factor; ZEB2, zinc finger E‐box binding homeobox 2.

### 3.1 Classical DCs

cDCs, which originate from myeloid progenitors, comprise two principal subsets: cDC1s and cDC2s. cDC1s are characterized by the specific expression of the Ca^2^⁺‐dependent (C‐type) lectin receptor (CLR) CLEC9A and the chemokine receptor XCR1 [129], whereas cDC2s express the highly conserved transmembrane protein SIRPα [130].

#### 3.1.1 Classical DC1s

cDC1s are a relatively scarce subset derived from CDPs, whose abundance is approximately one‐tenth that of cDC2s. They specialize in cross‐presenting antigens to CD8⁺ T cells and are key initiators of antitumor immunity [131, 132]. Their development and functional maturation depend on key transcription factors, including IRF8, NFIL3, BATF3, PU.1, and ID2 (Figure 2) [99, 100, 101]. Human cDC1s were initially identified as a blood DC subset expressing high levels of CD141 (BDCA‐3, thrombomodulin). They also highly express MHC class I and II molecules and the costimulatory molecule CD40, and moderate levels of CD11c (significantly lower than those in cDC2s) and key functional receptors [133]. The C‐type lectin receptor CLEC9A (DNGR‐1) is a highly conserved and specific marker for cDC1s. It recognizes actin filaments exposed by necrotic cells and functions as a damage sensor that channels cell‐associated antigens into the cross‐presentation pathway [134, 135]. Notably, CLEC9A expression exhibits tissue heterogeneity (e.g., lower levels in tonsils and skin) and can be affected by isolation methods such as collagenase treatment. Another conserved marker is the chemokine receptor XCR1, which mediates critical interactions between cDC1s and activated CD8⁺ T cells or NK cells expressing its ligand, XCL1/XCL2 [136]. Interestingly, the cDC1 population exhibits bimodal XCR1 expression. The XCR1^−^ subset may represent an immature or tissue‐resident state with the potential to proliferate and differentiate into XCR1⁺ cDC1s [137, 138].

cDC1s possess an exceptional antigen‐presenting capacity. They efficiently capture exogenous antigens (from viruses, intracellular pathogens, or dead/tumor cells) and present them via MHC class I molecules to activate antigen‐specific CD8⁺ cytotoxic T lymphocytes (CTLs) [102, 103]. This cross‐presentation ability underpins their role in antitumor immunity, as CTLs can directly kill tumor cells [139]. Although cDC1s are scarce, their abundance in tumors correlates with an improved prognosis and increased overall survival of individuals with various cancers, highlighting their crucial roles in antitumor immune responses and immunotherapy [140]. The maturation of cDC1s into fully functional, tumoricidal cells capable of recruiting and activating tumor‐specific T cells requires NF‐κB signaling, IKKβ, and IRF1 [141]. Their migration and infiltration into tumors, a process regulated by chemokines (e.g., CCL5 and XCL1 secreted by NK cells) in the TME, are vital for orchestrating local and lymphoid immune responses [142]. Recent studies have shown that CD4⁺ T cells can optimize cDC1 function. Through CD40L and IFN‐β, they support antigen cross‐presentation and T‐cell priming, inducing CTL‐mediated antitumor responses—a feature not observed with other DC types [143, 144]. CD4⁺ T cells can also induce CTL‐based antitumor immunity by producing IFN‐I [145]. IFN‐I enhances tumor‐associated antigen presentation, suppresses the activity of protumorigenic regulatory T cells (Treg cells) and/or myeloid‐derived suppressor cells (MDSCs), and enhances effector CD8⁺ T‐cell and Th1 function [145]. Furthermore, Hubert et al. [146] found that human cDC1s can mediate antitumor immunity by producing IFN‐III. cDC1s specifically produce IFN‐λ1, and this cytokine and its receptor are both associated with favorable patient outcomes. IFN‐III also increases IL‐12p70 and IFN‐γ levels, promotes chemokine‐mediated recruitment of cytotoxic lymphocytes, and induces the formation of a Th1‐polarized microenvironment.

#### 3.1.2 Classical DC2s

cDC2s are DCs that originate from CDPs and are pivotal for activating CD4⁺ T cells and orchestrating immunity against bacteria and fungi [112, 113]. Their development in humans relies on multiple transcription factors, including IRF4, KLF4, and NOTCH2 [106, 107, 108]. Unlike pDCs and cDC1s, no single transcription factor exclusively controls cDC2 development. For instance, while IRF4 is considered a key regulator, the heterozygous loss of GATA2 can also lead to a loss of cDC2s [147]. cDC2s are characterized by the expression of CD1c, CD11c, SIRPα, CD2, and FcεR1 and typically lack markers for cDC1s (e.g., CD141 and XCR1) or pDCs (e.g., CD123) [148]. They also express the myeloid antigens CD11b, CD13, and CD33 [148]. In tissues, skin cDC2s can be distinguished from LCs by higher CD11c and CD11b expression and lower CD1a, Langerin, and EpCAM expression [149]. Notably, cDC2s express different markers across tissues; for example, intestinal cDC2s highly express CD103, while those in the lungs and skin highly express CD1a [150]. Studies also indicate two subsets in human blood: one that is more “DC‐like” with higher levels of CD5, CD1c, HLA‐DQ, and IRF4 and another that is more “monocyte‐like” and expresses CD14, CD32, CD36, and CD163 and relatively higher levels of MAFB [109, 151].

Functionally, cDC2s are highly proficient in antigen processing and presentation, particularly in the regulation of CD4⁺ T‐cell responses [112, 113]. They widely express pattern recognition receptors (PRRs), such as TLR2, 4, 5, 6, 8, NOD‐like receptors, and RIG‐I‐like receptors (RLRs), which enable them to effectively recognize and respond to bacteria, fungi, viruses, and allergens. They produce large amounts of inflammatory cytokines (e.g., IL‐1β, IL‐6, IL‐23, and TNF‐α) and regulatory cytokines (e.g., IL‐10) [109, 110, 111]. Compared with cDC1s, human cDC2s are more readily induced to produce IL‐12 in vitro [109]. They efficiently induce the differentiation of Th1 cells, Th2 cells, Th17 cells, Th22 cells, and Treg cells, demonstrating their dual potential for immune activation and tolerance [109, 152]. cDC2 function exhibits significant heterogeneity. Based on CD5 expression levels, these cells can be divided into CD5‐high‐ and CD5‐low‐expressing subsets [153]. CD5‐high cDC2s are more active in C‐C chemokine receptor 7 (CCR7)‐dependent migration, potently stimulate naïve T‐cell proliferation, and preferentially induce Th2, Th17, and Treg cells responses [109, 154]. Conversely, CD5‐low cDC2s primarily induce Th1 cell differentiation [109, 154]. Human cDC2s can also perform antigen cross‐presentation to initiate CD8⁺ T‐cell responses [113]. They primarily present antigens via MHC class II to Th cells in secondary lymphoid organs in a subset‐dependent manner [11]. Given the important role of CD4⁺ T cells in tumor immune surveillance, cDC2s may also contribute to antitumor immunity [113].

### 3.2 Plasmacytoid DCs

pDCs represent a unique subset that directly originates from the Cx3cr1⁺ precursors of CDPs [155]. They exhibit a distinct morphology resembling antibody‐secreting plasma cells and lack the typical veiled dendrites of cDCs in the steady state. Additionally, pDCs possess several lymphocyte‐like features, including a lymphoid morphology, lymphatic circulation patterns, and the expression of lymphocyte markers such as CD45RA [156]. These characteristics once sparked debate regarding their lineage affiliation [157, 158]. Although both myeloid and lymphoid progenitors can differentiate into pDCs after transplantation or under in vitro culture conditions [55, 157, 158], which likely reflects their potential for pDC differentiation rather than their actual contribution to endogenous pDC development during hematopoiesis [156]. pDCs can be subdivided into CD2‐high and CD2‐low subsets, both of which exhibit significant activation and the ability to produce cytokines upon viral stimulation [121, 159].

pDCs play a crucial role in antiviral immunity, particularly in sensing viral infections and producing IFNs [19]. CD2 subsets produce IFN‐I. pDCs recognize various RNA and DNA viruses (including Vesicular Stomatitis Virus Glycoprotein, Hepatitis C Virus [HCV], Murine Cytomegalovirus, Hepatitis A Virus, Lymphocytic Choriomeningitis Virus, Dengue virus, West Nile virus, Epstein–Barr virus, Human Immunodeficiency Virus [HIV], and Murine Cytomegalovirus) through endosomal TLR7 and TLR9, leading to the secretion of IFN‐I (IFN‐α/β) [114]. TLR7 recognizes viral single‐stranded RNA (ssRNA), while TLR9 specifically recognizes DNA viruses and endogenous DNA containing unmethylated CpG sequences [115, 116]. This process involves the myeloid differentiation primary response protein 88 (MyD88)–IRF7 pathway for IFN production and the MyD88–NF‐κB pathway for the release of proinflammatory cytokines such as TNF and IL‐6 [117]. pDCs also secrete IFN‐III, which is particularly important in mucosal antiviral responses [19]. Interestingly, unlike the relatively uniform production of TNF‐α, IFN production is tightly regulated, with only a subset of pDCs producing IFNs [55]. Furthermore, if activated by appropriate TLR ligands or maturation stimuli (e.g., CD40L), pDCs can induce robust T‐cell activation and acquire an APC phenotype [138, 160]. The CD2‐high pDC subset secretes higher levels of IL‐12 p40 and promotes T‐cell proliferation more efficiently [124]. Overall, as frontline cells involved in antiviral immunity, pDCs not only directly respond to viral infections through IFN production but also enhance overall immune responses via cytokine secretion and T‐cell activation.

### 3.3 Monocyte‐Derived DCs

moDCs, also referred to as “inflammatory DCs,” originate from myeloid progenitors [119]. Strictly speaking, they are not a constitutive subset present under steady‐state conditions but rather differentiate from monocytes recruited into tissues under inflammatory conditions. Research has shown that moDCs in inflamed tissues exhibit unique molecular signatures, sharing features with both cDCs and inflammatory macrophages, such as CD14 and the mannose receptor (CD206) [121]. Additionally, moDCs express transcription factors involved in DC differentiation, such as ZBTB46, MAFB, and KLF4. Given the numerous shared characteristics among moDCs, cDCs, monocytes, and macrophages, accurately distinguishing them from other cell types in vivo remains challenging [161]. Phenotypically, moDCs share similar expression patterns of MHC‐II, CD11b, and CD11c with cDCs, but their monocytic origin can be identified by the expression of CD64 and Fc‐γ receptor 1 (FCγR1) [162]. Studies indicate that monocyte‐derived cells function primarily at inflammatory sites rather than migrating to lymph nodes [163]. Monocytes are the key precursors for moDCs [164]. Following inflammation or infection, circulating monocytes express GM‐CSF, M‐CSF, IL‐4, and other differentiation factors and chemokines. They are recruited into tissues and attracted to inflammatory sites, where they differentiate into moDCs [119]. Furthermore, moDCs synthesize and release TNF‐α and inducible nitric oxide synthase (iNOS), providing innate immune protection [118]. Consequently, these cells are also termed “TNFα‐ and iNOS‐producing DCs” [119].

Under inflammatory conditions, moDCs exhibit an activated phenotype, producing proinflammatory cytokines such as IL‐1, IL‐12, and IL‐23 and inducing the differentiation of CD4⁺ T cells into Th1 and Th17 cells, suggesting that these cells play key roles in autoimmune diseases and inflammatory responses [121]. Upon activation, moDCs have a lifespan similar to that of cDCs and exhibit highly efficient antigen processing, presentation, and cross‐presentation, enabling them to present antigens to T cells and initiate immune responses [60]. Moreover, moDCs have been detected in various pathological conditions, including atopic dermatitis, psoriasis, rheumatoid arthritis (RA), and tumor ascites. Their secretion of IL‐23, which is associated with tumor growth, makes them potential targets for antitumor immunotherapy [120].

### 3.4 Langerhans Cells

LCs are a unique population of mononuclear phagocytes residing within stratified epithelial tissues. When classified as a subset of DCs derived from macrophage progenitors, LCs possess a typical veiled morphology and long dendrites [122]. They primarily originate from the differentiation of MPs; concurrently, conventional cDC2s and monocytes expressing high levels of Gr‐1 can also migrate to the epidermis and differentiate into LCs within the local microenvironment [122]. LCs are located mainly in the basal/suprabasal layers of stratified epithelial tissues, such as the epidermis, urogenital/oral mucosa, and cornea [165]. Their development depends on IL‐34, a cytokine secreted mainly by neurons and keratinocytes, which supports their differentiation and maintenance into adulthood. As professional immune sentinels in the epidermis, LCs form the first line of immune defense. Their characteristic veiled dendrites continuously monitor the epidermal microenvironment, penetrating tight junctions to directly capture antigens (e.g., pathogens or commensals) while maintaining the immunoprotective network without compromising skin barrier integrity [166]. LCs migrate to lymph nodes under both steady‐state and inflammatory conditions to initiate immune responses. They detach from keratinocytes by downregulating the expression of molecules such as E‐cadherin, upregulate the expression of MHC II, present antigens to T cells, and induce humoral immunity [167]. Studies have shown that under homeostatic conditions, LCs promote epidermal health and immune tolerance, maintaining tolerance to commensals [168]. Under inflammatory conditions, these cells retain the ability to respond to intracellular pathogens and viruses and can induce CTL responses by secreting IL‐15 [123, 124]. Two other studies also indicated that LCs can mature into potent cross‐presenting DCs with a high IL‐15 production capacity, presenting mycobacterial glycolipid antigens and activating CD8⁺ T cells [125, 126].

LCs exhibit a cross‐presentation ability, express high levels of MHC class I‐related genes, and interact with T cells expressing langerin, CD1a, and CD1c in lymph nodes [109]. During skin inflammation, locally produced TNF‐α and IL‐1β can stimulate LCs to cross the basement membrane into afferent lymphatics. However, LCs lack key TLRs and can induce the production of Treg cells (Th22 cells) and IL‐22 through CD1a‐restricted antigens [127]. Furthermore, LCs can induce inflammatory responses through the polarization of helper T cells into different subsets that respond to intracellular pathogens (Th1 cells) and parasitic worms (Th2 cells), and by promoting the production of Th17 cells, which are involved in autoimmune and inflammatory diseases [122]. LCs are also associated with various pathologies. For instance, they regulate the nerve fiber density in the epidermis and dermis by secreting neurotrophic factors (e.g., NGF and GDNF), and their dysfunction may be linked to sensory neuropathy [122]. Additionally, LCs play a key role in the pathophysiology of atopic dermatitis [128]. In summary, LCs act as a central hub in skin barrier defense and homeostatic regulation by dynamically balancing tolerance and immune activation.

In summary, cDC1s, cDC2s, pDCs, moDCs, and LCs constitute the core subsets of DCs. In response to specific microenvironmental signals (e.g., IL‐10, TGF‐β, vitamin D3, glucocorticoids, or exposure to apoptotic cells), these DC subsets may transform into tolDCs, also known as regulatory DCs. These cells induce and maintain immune tolerance, preventing autoimmune responses by reducing the antigen presentation capability, decreasing immune activation, and secreting inhibitory cytokines. In contexts such as chronic inflammation, organ transplantation, allergic reactions, and tumor immune evasion, these transformed cell populations play important roles in helping the immune system avoid excessive responses or self‐attack. Therefore, the formation and function of tolDCs are important for immune homeostasis, immunotherapy, and the prevention and treatment of immune diseases.

## 4 Biological Functions of DCs

The immune functions of DCs are initiated by the capture of antigens in peripheral tissues, triggering a cascade of sophisticated biological processes. This section comprehensively describes the functional cascade of DCs, from classical antigen capture, processing, and presentation, through migration to lymphoid organs, to the ultimate activation of naïve T cells, regulation of B‐cell responses, induction of immune tolerance, and interactions with innate immune cells such as NK cells, thereby underscoring their pivotal role as the central orchestrators of the immune system.

### 4.1 DC Antigen Capture, Maturation, and Migration

**FIGURE 3:**
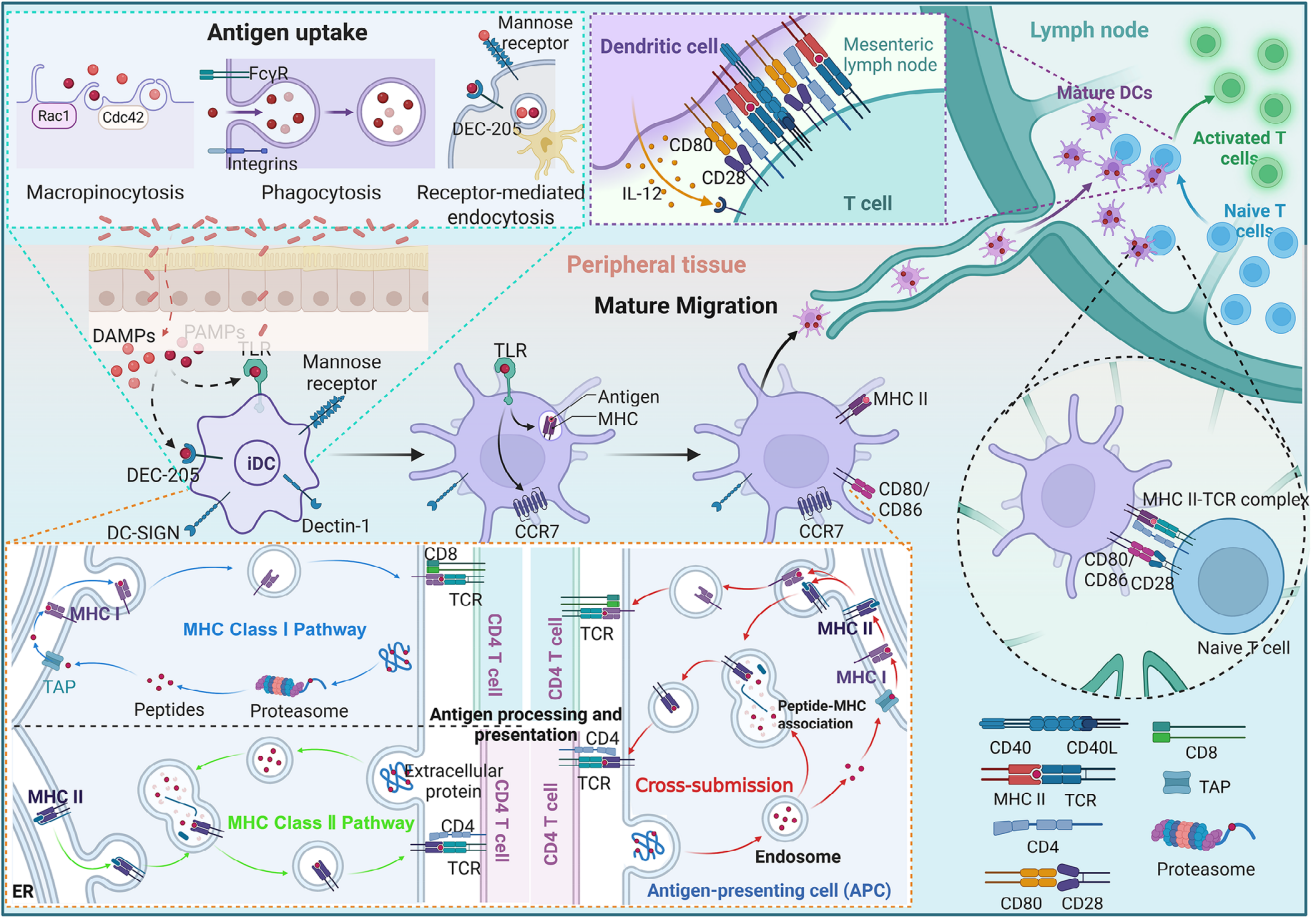
The processes of antigen capture, maturation, migration, antigen processing and presentation in dendritic cells. This schematic diagram integrates all the functions of dendritic cells (DCs), from antigen capture, maturation, and migration to antigen processing and presentation. Immature DCs (iDCs) capture antigens in peripheral tissues through macropinocytosis (regulated by Rac1/Cdc42), phagocytosis (mediated by FcγR/integrin), and receptor‐mediated endocytosis (involving DEC205/mannose receptors). They subsequently mature in response to PAMPs/DAMPs (such as LPS) and inflammatory cytokines (such as TNF/IL‐1). These factors upregulate the expression of MHC‐II, costimulatory molecules (CD80/86), and the chemokine receptor CCR7; in addition, these cells secrete IL‐12/TNF, adopt a dendritic morphology, and express fascin‐1 to increase migration. These DCs then migrate to lymph nodes via lymphatic vessels in response to a CCL19/CCL21 gradient. In terms of antigen processing, endogenous antigens are degraded by proteasomes and then transported to the endoplasmic reticulum by TAP, loaded onto MHC‐I and presented to CD8⁺ T cells. Exogenous antigens are degraded in endosomes/lysosomes (through the action of tissue proteases) and loaded onto MHC‐II for presentation to CD4⁺ T cells. The cDC1 subset is the core cross‐presenting subset, which recognizes necrotic cell antigens through CLEC9A and triggers the rupture of phagosomes, allowing exogenous antigens to enter the cytoplasmic MHC‐I pathway (or be directly loaded into the endocytic cavity). Its efficient cross‐presentation ability is due to low protease activity and the assistance of molecules such as WDFY4, while cDC2s and moDCs have only a limited cross‐presentation ability. *Abbreviations*: iDCs, immature dendritic cells; PAMPs, pathogen‐associated molecular patterns; DAMPs, damage‐associated molecular patterns; LPS, lipopolysaccharide; TNF, tumor necrosis factor; IL, interleukin; TAP, transporter associated with antigen processing.

Typically, immature DCs (iDCs) possess a robust capacity for antigen capture. Residing in peripheral tissues (e.g., skin, mucosa, and nonlymphoid organs), they collectively become activated and migrate to draining lymph nodes in the presence of pathogens. Concurrently, monocytes are recruited from peripheral blood into inflamed tissues, where they rapidly differentiate into antigen‐capturing iDCs. iDCs rely on highly expressed PRRs, such as CLRs, Toll‐like receptors, and Fc receptors, to recognize and internalize diverse antigens, including bacteria, viruses, apoptotic cells, and soluble proteins [169]. Antigen capture is subsequently achieved through macropinocytosis, phagocytosis, and receptor‐mediated endocytosis (Figure 3) [169]. Macropinocytosis is an actin‐dependent, large‐scale form of endocytosis. Through extensive membrane ruffling or protrusions, cells nonspecifically internalize extracellular fluid, soluble molecules, nutrients, and antigens into large vesicles known as macropinosomes [170]. This process occurs constitutively in DCs, endowing them with a high‐capacity, nonsaturable mechanism for capturing virtually any soluble antigen, which constitutes a powerful nonspecific antigen uptake pathway [171, 172]. The small GTPases Rac1 and Cdc42, which regulate actin polymerization, are critical regulators of constitutive macropinocytosis in DCs [173]. Phagocytosis is a specialized mechanism for the cellular uptake of large solid particles, such as bacteria, viruses, dead cells, and other large particulate matter, via vesicle formation [174]. It is performed primarily by professional phagocytes such as macrophages and DCs [174]. Phagocytic uptake occurs through a receptor‐engaged “zipper” mechanism involving various receptors, including integrins, FcγRs, and scavenger receptors [174]. Receptor‐mediated endocytosis facilitates the highly efficient uptake of low‐concentration antigens through specific cell surface receptors. Unlike macropinocytosis, it is a specific, receptor‐guided process. DCs express several C‐type endocytic lectins, such as DEC205, the mannose receptor, and Langerin, as well as type I and II Fcγ receptors [171, 175, 176, 177]. After capturing antigens, DCs receive maturation signals from pathogen‐associated molecular patterns (PAMPs), damage‐associated molecular patterns (DAMPs), or inflammatory mediators, including LPS, viral RNA, signals released by necrotic cells, TNF, IL‐1, IL‐6, IL‐10, and TGF‐β. These stimuli drive the transition from an immature state to a mature state. During maturation, the surface expression of MHC molecules and costimulatory molecules (CD80, CD83, and CD86) is markedly upregulated [178]. Moreover, DCs begin secreting cytokines such as IL‐12, IL‐10, and TNF, further amplifying immune responses [178]. Morphologically, maturing DCs transition from a round, smooth appearance to a rough morphology with multiple dendrites or pseudopods. Mature DCs upregulate the expression of the chemokine receptor CCR7, enabling them to respond to the lymph node‐homing chemokines CCL19 and CCL21. The expression of CCR7, coupled with inflammatory mediators (e.g., prostaglandin E2) at the site of infection, allows DCs to egress from the inflamed tissue and migrate to the T‐cell zones of draining lymph nodes. Furthermore, mature DCs express higher levels of fascin‐1, an actin‐bundling protein that increases their migratory capacity. Utilizing active movement of their dendrites, they can transit to lymph nodes more rapidly than their immature counterparts (Figure 3).

### 4.2 Antigen Processing and Presentation by DCs

iDCs possess a potent antigen‐processing capacity [179]. Platt and colleagues [180] further demonstrated that although macropinocytosis is markedly downregulated upon DC maturation, mature DCs can still acquire and process antigens through receptor‐mediated endocytosis and phagocytosis. Following capture by DCs, antigens are degraded into peptides within specific cellular organelles. These peptides are then precisely loaded onto MHC molecules to form pMHC complexes, which are subsequently transported to the cell surface for recognition by T cells (Figure 3) [181]. DCs employ distinct pathways depending on the origin of the antigen. Endogenous antigens, such as intracellular proteins produced during viral infection, are degraded by proteasomes into short peptides in the cytosol. These peptides are translocated into the endoplasmic reticulum (ER) via the transporter associated with antigen processing (TAP), where they bind nascent MHC class I molecules to generate pMHCI complexes. These complexes are subsequently trafficked through the Golgi apparatus to the plasma membrane for presentation to CD8⁺ T cells, initiating cytotoxic T‐cell responses (Figure 3) [182]. In contrast, exogenous antigens, including phagocytosed bacteria, viral particles, extracellular proteins, or apoptotic cells, enter the endosomal–lysosomal system via endocytosis or phagocytosis. In the acidic milieu, these antigens are degraded by proteolytic enzymes such as cathepsins into peptides that associate with MHC class II molecules within late endosomes or MHC class II compartments. The resulting pMHCII complexes are then transported to the surface for recognition by CD4⁺ T cells (Figure 3) [183]. The differentiation of CD4⁺ T cells into effector subsets (Th1, Th2, Th17, and Tfh) is finely tuned by the nature of the captured antigen, the repertoire of costimulatory molecules expressed by DCs (e.g., CD40, CD80, and CD86), and the cytokines they secrete (e.g., IL‐12, IL‐4, IL‐6, and IL‐23) [119]. A defining feature of DC biology is their cross‐presentation ability, whereby exogenous antigens are presented on MHC class I molecules to activate CD8⁺ T cells [184]. This process is essential for immune defenses against tumors and intracellular pathogens. Among cDCs, the cDC1 subset is the primary executor of cross‐presentation and is particularly adept at capturing and processing antigens derived from apoptotic or necrotic cells (e.g., tumor‐associated antigens), thereby efficiently priming CD8⁺ T‐cell responses [184, 185]. Cross‐presentation largely occurs through two TAP‐dependent cytosolic routes (Figure 3): (1) internalized antigens escape from endocytic/phagocytic vesicles into the cytosol, where they enter the proteasome–TAP–ER–MHC I pathway; and (2) antigen peptides are directly loaded onto MHC I molecules within endocytic compartments where TAP and the related machinery are recruited [185]. Notably, the cDC1‐specific receptor CLEC9A/DNGR1 recognizes exposed actin filaments from necrotic cells, facilitating both antigen uptake and phagosomal rupture, thus promoting the cytosolic entry of antigens for cross‐presentation [186]. Additionally, molecules such as WDFY4 are involved in intracellular antigen trafficking to promote cross‐presentation [187]. DCs also adopt specialized mechanisms to preserve antigen integrity for cross‐presentation, including the maintenance of a higher endosomal pH, reduced protease activity, and expression of protease inhibitors [188]. Although cDC2s are traditionally viewed as specialized for MHC class II presentation, certain activation states (e.g., IFN‐α stimulation) or moDCs recruited to inflamed tissues may also acquire a cross‐presenting capacity [189, 190, 191]. In summary, DCs orchestrate antigen processing through tightly regulated degradation and highly specialized MHC loading pathways. Their unique ability to cross‐present exogenous antigens on MHC class I molecules significantly broadens their surveillance capacity. By precisely presenting processed antigens as pMHC complexes, DCs provide the antigen‐specific signals required for T‐cell activation, clonal expansion, and differentiation, thereby playing a central role in initiating and regulating adaptive immunity.

### 4.3 Activation of Adaptive Immunity

**FIGURE 4:**
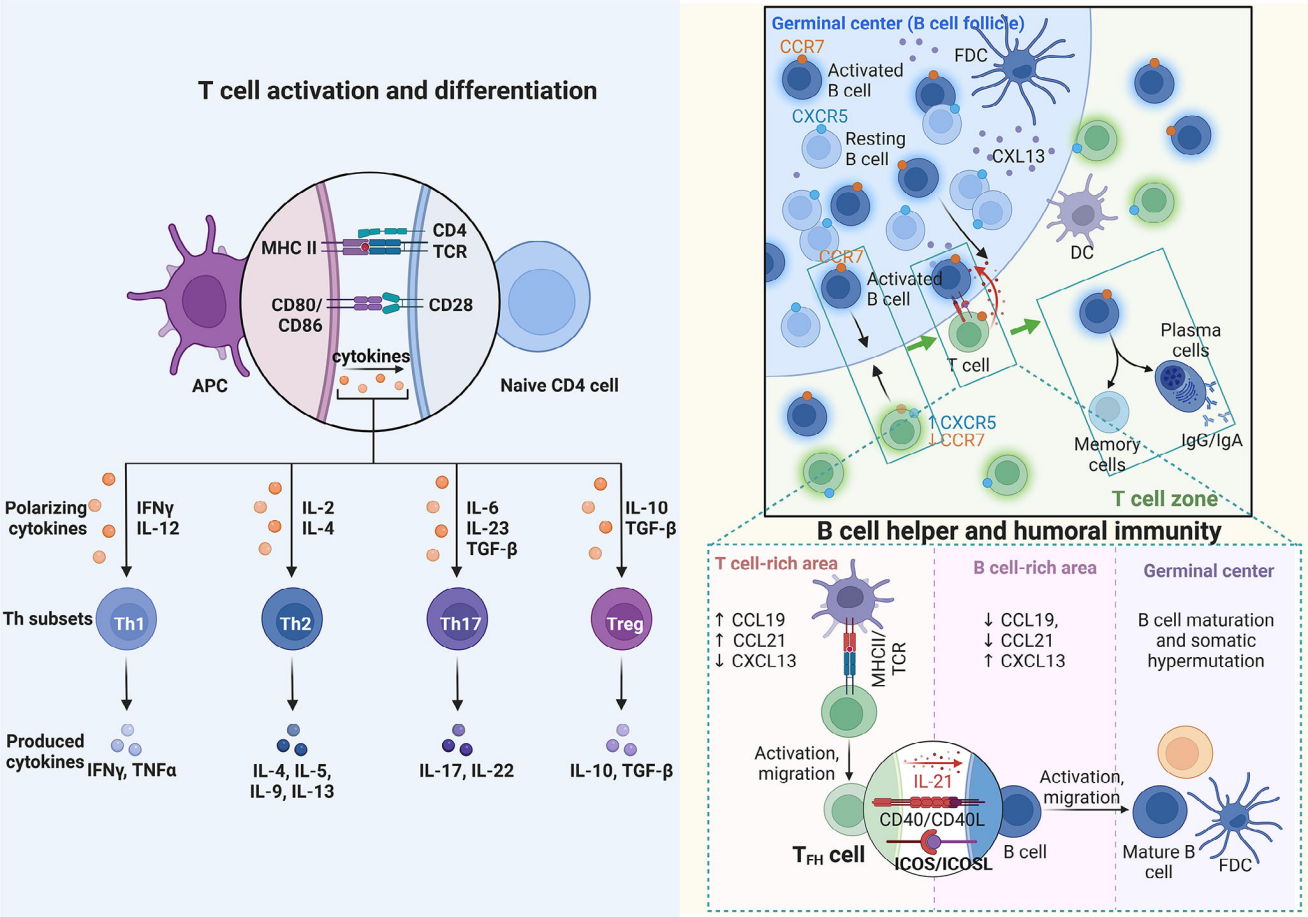
Dendritic cells initiate the core process of the adaptive immune response. This diagram illustrates the core process by which dendritic cells (DCs) initiate adaptive immune responses: mature DCs that migrate to secondary lymphoid organs activate naïve T cells through a three‐signal mechanism, namely, TCR recognition of pMHC, CD80/CD86 binding to CD28 to provide costimulation, and the secretion of cytokines such as IL‐12, thereby guiding CD4⁺ T cells to differentiate into Th1 (T‐bet⁺, secreting IFNγ, TNFα), Th2 (GATA3⁺, secreting IL‐4/5/9/13), Th17 (RORγt⁺, secreting IL‐17/22), regulatory T cells (Treg, FoxP3⁺, secreting IL‐10). Tfh cells migrate to B‐cell follicles by downregulating CCR7 and overexpressing CXCR5. With the help of TCR–pMHCII recognition, CD40L–CD40 binding and the ICOS–ICOSL interaction, and synergistic interactions with IL‐4/IL‐21, they activate B cells to initiate germinal center reactions, causing them to undergo high‐frequency somatic cell mutations, affinity maturation and antibody class conversion (such as conversion from IgM to IgG/IgA), and finally differentiate into long‐acting plasma cells (continuously secreting high‐affinity antibodies) and memory B cells, thereby bridging and regulating efficient humoral immune responses. *Abbreviations*: TCR, T‐cell receptor; pMHC, peptide–major histocompatibility complex; Tfh, T follicular helper; IL, interleukin; IFNγ, interferon gamma; TNFα, tumor necrosis factor alpha; Treg, regulatory T; IgG, immunoglobulin G; IgA, immunoglobulin A; IgM, immunoglobulin M.

After migrating to secondary lymphoid organs, DCs perform their central function of activating naïve T cells, a critical step in initiating adaptive immune responses [185, 192]. T‐cell activation depends on three major signals [193]: (1) the recognition of peptide–MHC complexes on DCs by the T‐cell receptor (TCR), (2) the engagement of costimulatory molecules such as CD80 with TCRs, including CD28 or CTLA‐4, and (3) the secretion of cytokines (e.g., IL‐12, type I IFNs, and IL‐4) by activated DCs. The first two signals establish an activation threshold, whereas the third guides lineage differentiation (Figure 4) [193]. Naïve CD4⁺ T cells can differentiate into diverse effector subsets, including Th1, Th2, Th17, Tfh, and Treg cells (Figure 4) [193, 194]. IL‐12 and IFN‐γ induce T‐bet‐dependent Th1 differentiation [193, 195]; IL‐4 promotes Th2 polarization [196, 197]; TGF‐β together with IL‐6, IL‐1β, IL‐21, or IL‐23 drives Th17 differentiation [198, 199]; and IL‐6, IL‐21, IL‐12, and IL‐23 promote Tfh differentiation [193, 200], whereas TGF‐β induces Treg cell development [201]. Each subset performs distinct functions: Th1 cells mediate cellular immunity by secreting IL‐12, IFN‐γ, and TNF‐α to activate macrophages and enhance CTL responses, which are critical to protect against intracellular pathogens [202, 203, 204]. Th2 cells secrete IL‐4, IL‐5, IL‐9, and IL‐13 to support antibody production (especially IgE production), contributing to antiparasitic defenses and allergic responses [196, 205]. Th17 cells produce IL‐17 and IL‐22, recruiting neutrophils to clear extracellular bacteria and fungi while maintaining mucosal homeostasis [206, 207]. Tfh cells promote class switching and high‐affinity antibody responses and play pivotal roles in humoral immunity and autoimmune and allergic diseases [200]. In addition, Tfh cells support B‐cell‐mediated humoral immune responses through the expression of CD40L and the secretion of IL‐21 (Figure 4) [200]. Treg cells suppress immune activation by secreting IL‐10 and TGF‐β, thereby maintaining tolerance [203]. With the help of CD4⁺ T cells, CD8⁺ T cells can differentiate into CTLs. CTLs directly eliminate infected and tumor cells via the release of perforin/granzymes or through the FasL/Fas pathway [208]. They also secrete IFN‐γ to amplify overall immune responses [209]. A portion of activated T cells ultimately differentiates into memory T cells, including circulating memory T cells and nonrecirculating tissue‐resident memory T cells [210]. Compared with naïve T cells, memory T cells persist at a higher frequency and exhibit a lower activation threshold [211]. Upon re‐encountering the same pathogen, they mount a more rapid, robust, and effective secondary immune response, enabling swift pathogen clearance and providing long‐term immunological protection [211].

As mentioned, DC‐mediated T‐cell activation promotes Tfh cell differentiation. Tfh cells are characterized by high expression of C‐X‐C chemokine receptor 5 (CXCR5), CD40L, inducible T‐cell costimulator (ICOS), and programmed death‐1 (PD‐1) and low expression of CCR7 [200, 212, 213]. The downregulation of CCR7 allows CXCR5⁺ Tfh cells to migrate into B‐cell follicles, where they encounter B cells [200, 212, 213]. Within the follicle, the TCR on Tfh cells recognizes the pMHCII complex on B cells. The subsequent engagement of CD40L (on Tfh cells) with CD40 (on B cells) is essential for full B‐cell activation, proliferation, germinal center formation, and antibody class switching [213]. ICOS–ICOSL engagement provides further costimulation, while the Tfh‐derived cytokines IL‐4 and IL‐21 directly promote B‐cell differentiation (Figure 4) [200, 214, 215]. With this help from Tfh cells, activated B cells proliferate and form germinal centers [200]. Within this specialized microenvironment, B cells undergo somatic hypermutation, affinity selection mediated by follicular DCs, and Tfh‐dependent class switching [216, 217, 218, 219]. These processes ultimately give rise to long‐lived plasma cells, which secrete high‐affinity IgG/IgA antibodies, and memory B cells (Figure 4) [216, 217, 218, 219]. Plasma cell‐derived antibodies neutralize pathogens, opsonize microbes, activate complement, and mediate ADCC, whereas memory B cells ensure a rapid and robust secondary humoral response [220]. In summary, by activating the Tfh cell population, DCs indirectly bridge innate and adaptive humoral immunity, serving as central regulators in the initiation of high‐efficiency, specific, and durable antibody responses.

### 4.4 Regulation of Immune Tolerance

**FIGURE 5:**
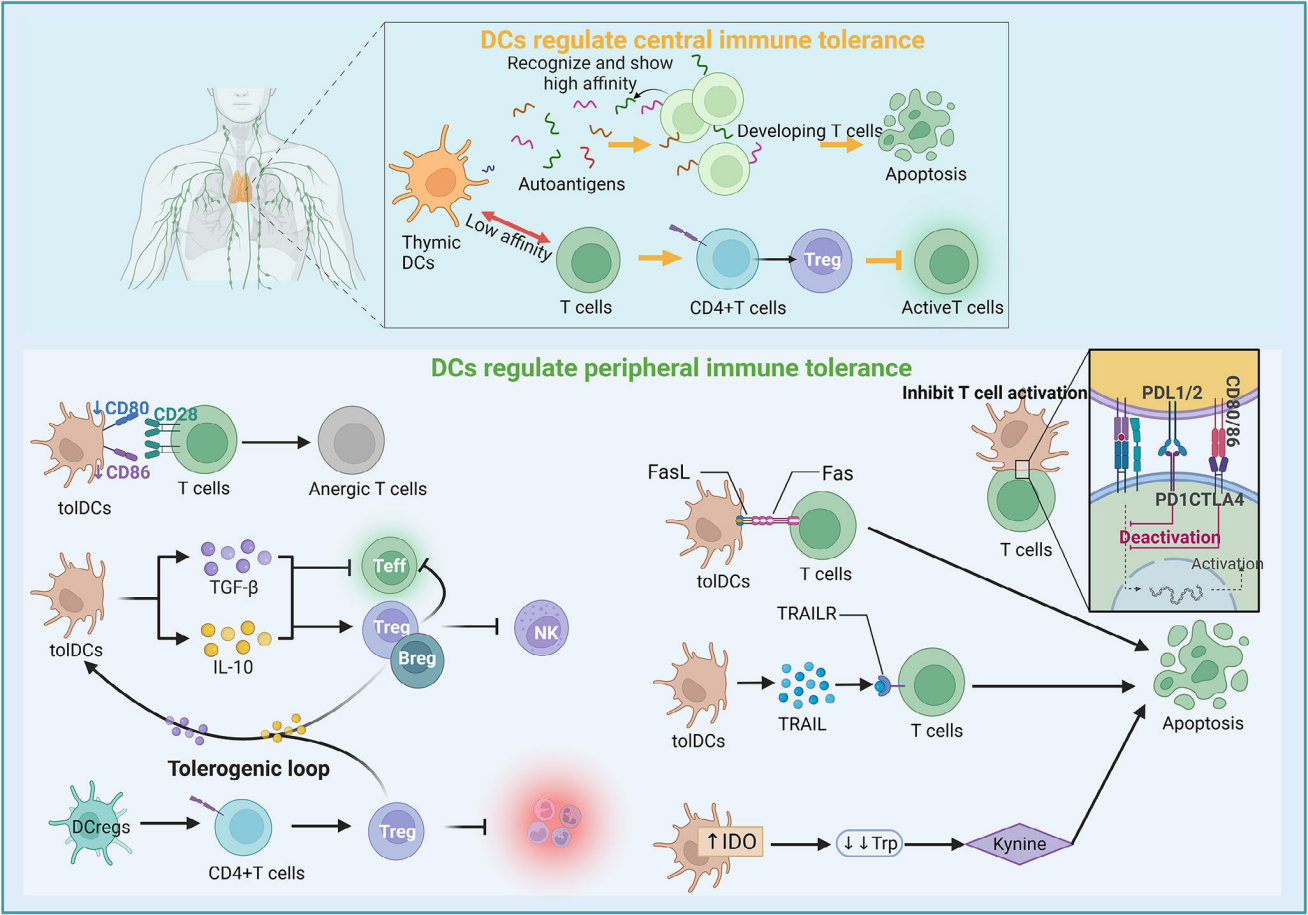
DCs maintain immune tolerance through both central and peripheral mechanisms. This schematic diagram illustrates how dendritic cells (DCs) maintain immune tolerance through both central and peripheral mechanisms. In the thymus, thymic DCs mediate central tolerance by presenting self‐antigens, inducing the apoptosis of high‐affinity autoreactive T cells (negative selection) on the one hand, and guiding low‐affinity CD4⁺ T cells to differentiate into Treg cells to inhibit effector T‐cell activation on the other hand. In the periphery, tolerogenic DCs (tolDCs) cause initial T‐cell dysfunction through the low expression of costimulatory molecules and inhibit T‐cell activation and proliferation through multiple pathways, such as the secretion of IL‐10/TGF‐β and high expression of immune checkpoint molecules (PD‐L1/CTLA‐4 ligands), metabolic enzymes (IDO depletion of tryptophan and HO‐1 produces carbon monoxide), and death ligands (TRAIL/FasL mediate T‐cell apoptosis), while inducing Treg expansion and regulatory B‐cell generation, thereby forming an immunosuppressive positive feedback loop and ultimately establishing and maintaining long‐term immune tolerance. *Abbreviations*: IL, interleukin; TGF‐β, transforming growth factor beta; PD‐L1, programmed death‐ligand 1; CTLA‐4, cytotoxic T‐lymphocyte‐associated protein 4; IDO, indoleamine 2,3‐dioxygenase; HO‐1, heme oxygenase 1; TRAIL, TNF‐related apoptosis‐inducing ligand; FasL, Fas ligand.

Immunological tolerance refers to a state in which the immune system does not mount excessive inflammatory responses to self‐antigens or harmless foreign antigens (e.g., food proteins, commensal microbiota, and transplanted organs). DCs maintain immune tolerance through dual central and peripheral mechanisms (Figure 5) [119, 221]. Central tolerance occurs in the thymus [119]. Thymic DCs contribute to immune tolerance by mediating negative selection and inducing Treg cells [221]. Studies have shown that thymic DSCs express large amounts of autoantigens [221, 222]. When developing T cells recognize these autoantigens and exhibit high affinity, they undergo apoptosis (Figure 5) [221, 222]. Additionally, thymic DCs can interact with T cells with lower affinity, guiding certain self‐reactive CD4⁺ T cells to differentiate into Treg cells [221, 222]. These Treg cells function to suppress the activation of autoreactive effector T cells. Peripheral tolerance is largely mediated by tolDCs through multiple mechanisms (Figure 5). First, tolDCs express low levels of costimulatory molecules. When antigens are presented to naïve T cells in the absence of sufficient costimulation, this incomplete activation signal drives T cells into a functionally inactivated state known as anergy [223]. Anergic T cells are unable to proliferate, differentiate into effector cells, or produce cytokines effectively, resulting in the lack of antigen‐specific responses. Second, tolDCs secrete immunosuppressive cytokines such as IL‐10 and TGF‐β, which inhibit effector T‐cell activation and proliferation [224]. Furthermore, research has demonstrated that tolDCs can promote the conversion of CD4⁺ T cells into Treg cells and support the expansion and functional maintenance of existing Treg cell populations [224]. In addition, tolDCs highly express inhibitory immune checkpoint molecules such as PD‐L1, PD‐L2, and ligands for CTLA‐4. The engagement of these molecules with their respective receptors (e.g., PD‐1 and CTLA‐4) on T cells directly suppresses T‐cell activation [225, 226]. These molecules also upregulate indoleamine 2,3‐dioxygenase (IDO), which depletes tryptophan and produces kynurenine, thereby inducing T‐cell apoptosis and promoting Treg cell differentiation [224, 227]. Similarly, tolDCs express heme oxygenase‐1 (HO‐1), generating carbon monoxide that modifies the metabolic milieu, reduces their immunogenicity, and suppresses T‐cell responses [227]. tolDCs can directly eliminate T cells via clonal deletion. Studies indicate that tolDCs express TRAIL and FasL, which engage corresponding death receptors on T cells, thereby inducing T‐cell apoptosis [228, 229]. Finally, tolDCs can induce Treg cells and regulatory B cells (Breg cells) to produce immunosuppressive factors such as IL‐10, IL‐35, and TGF‐β [230, 231]. This process promotes the conversion of other DCs toward a tolerogenic phenotype, thereby perpetuating a state of immune tolerance.

### 4.5 Regulation of Innate Immune Cells

**FIGURE 6:**
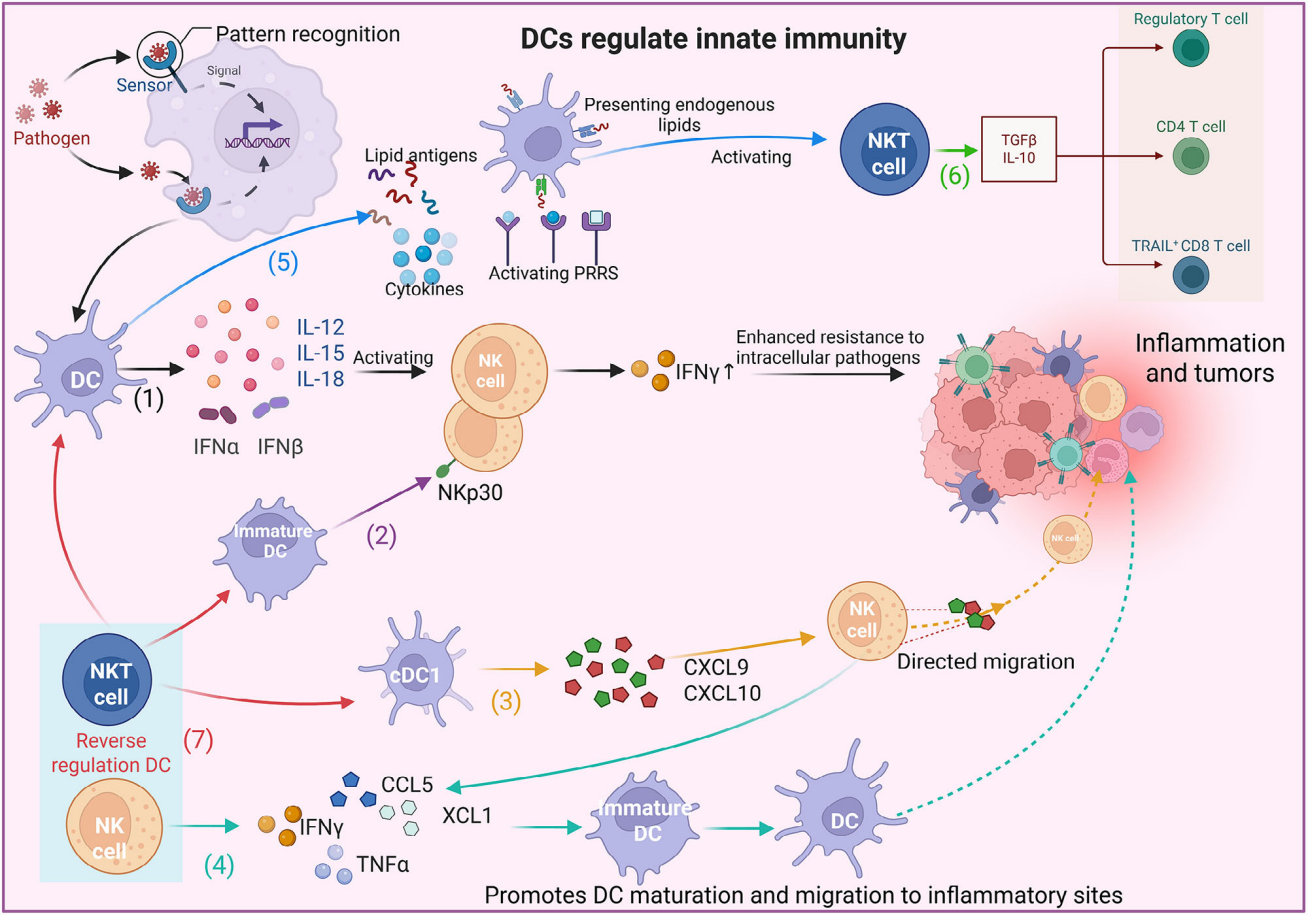
Dendritic cells orchestrate innate immunity through bidirectional crosstalk with NK and NKT cells. This schematic illustrates that DCs orchestrate innate immune responses via two primary dialog partners: NK cells and NKT cells. (1) DC‐derived soluble mediators (e.g., IL‐12, IL‐15, IL‐18, type I IFNs) prime NK cell cytotoxicity and IFN‐γ production. (2) Cell‐contact‐dependent signaling via NKp30 further activates NK cells. (3) DC‐secreted chemokines such as CXCL9 and CXCL10 recruit NK cells to target sites. (4) In feedback, activated NK cells enhance DC maturation and migration via IFN‐γ, TNF‐α, XCL1, and CCL5. (5) Concurrently, DCs activate NKT cells through direct lipid antigen presentation, cytokine secretion, or PRR‐mediated endogenous lipid presentation. (6) Activated NKT cells modulate DC phenotype, cytokine profile, and subset distribution, influencing subsequent T‐cell responses. (7) NKT cells also exert negative feedback on DCs, fine‐tuning overall immune activation. *Abbreviations*: NK, natural killer; IL, interleukin; IFN, interferon; CXCL, C‐X‐C motif chemokine; TNF, tumor necrosis factor; XCL1, chemokine (C motif) ligand 1; CCL5, chemokine (C‐C motif) ligand 5; PRR, pattern recognition receptor.

As the central link between innate and adaptive immunity, DCs orchestrate early immune responses by regulating the activity of NK cells and NKT cells through multiple mechanisms (Figure 6). First, DCs secrete cytokines such as IL‐12, IL‐15, IL‐18, and type I IFNs, which directly activate the cytolytic function of NK cells and induce an IFN‐γ burst, thereby enhancing defenses against intracellular pathogens [11]. Second, iDCs can activate NK cells via the NKp30 signaling pathway, promoting their proliferation and increasing their cytotoxicity to an extent comparable to that of IL‐2 [232]. Furthermore, cDC1s secrete chemokines, including CXCL9 and CXCL10, which guide the targeted migration of NK cells to sites of infection or tumors [104, 105]. NK cells activated by DCs reciprocally modulate DC function. Activated NK cells secrete substantial amounts of IFN‐γ, TNF‐α, and chemokines such as XCL1 and CCL5, which promote DC maturation and migration toward inflammatory sites [136, 233, 234]. Interestingly, NK cells can also identify and eliminate inadequately matured or dysfunctional DCs, ensuring that only those DCs passing this “quality control” process participate in subsequent adaptive immune responses [235]. A bidirectional regulatory relationship also exists between DCs and NKT cells. DCs activate NKT cells through several routes, direct presentation of microbial lipid antigens, secretion of cytokines, or the presentation of endogenous lipids following PRR activation, thereby efficiently inducing NKT cell activation and polarization [15]. In turn, NKT cells modulate DCs. Studies have demonstrated that NKT cells significantly influence the phenotype, cytokine profile, subset distribution, and function of DCs in the spleen and lungs, consequently shaping CD4⁺ and CD8⁺ T‐cell responses and antibody isotypes [236, 237, 238]. NKT cells can also negatively regulate DCs. For example, in an experimental autoimmune encephalomyelitis (EAE) model, type II NKT cells drive DCs into a tolerogenic state, suppressing pathogenic T‐cell responses [15, 239]. Furthermore, the impact of NKT cells on DCs is highly dependent on the microenvironmental context. In the absence of TLR4 costimulation, NKT cells may induce DC tolerance via the ERK1/2 pathway, whereas TLR4 costimulation can shift this effect toward a proinflammatory outcome [15, 240].

In summary, DCs establish a cellular and molecular foundation for immune responses and homeostatic balance through their sophisticated antigen processing and presentation mechanisms, effective initiation of adaptive immunity, and precise regulation of innate immune cells. These functions not only provide a critical theoretical and targeting basis for interventions in various immune‐mediated diseases but also shows the broad application prospects for immunotherapeutic strategies targeting DCs due to the functional diversity and plasticity of these cells.

## 5 Therapeutic Potential of DCs

Leveraging the central regulatory role of DCs in immune responses, therapeutic strategies targeting these cells have become a major research focus in oncology, diabetes, infectious diseases, transplantation, autoimmune disorders, cardiovascular diseases, allergic conditions, and dermatology. This section explores the dual roles of DCs (promoting or suppressing immunity) across different disease contexts and reviews DC‐based therapeutic strategies, including recent preclinical and clinical advances, while assessing their therapeutic potential and challenges.

### 5.1 DCs and Cancer

The core of tumorigenesis lies in the accumulation of genetic mutations driven by both genetic and environmental factors, leading to oncogene activation and the inactivation of tumor suppressor genes. These alterations endow tumor cells with a sustained proliferative capacity, resistance to apoptosis, ability to induce angiogenesis, and metastatic potential. However, a key determinant of successful tumor progression is the ability to evade immune surveillance. DCs are central orchestrators of antitumor immunity, and multiple studies have confirmed their crucial role in cancer immunity [132, 134, 241]. Their primary function is to capture, process, and cross‐present tumor‐associated antigens while they release costimulatory molecules and proinflammatory cytokines, thereby effectively priming T‐cell responses. Among DC subsets, cDC1s are crucial for antitumor immunity and produce IL‐12 to polarize naïve CD4⁺ T cells toward Th1 cells and subsequently activate CD8⁺ T cells [132]. CD4⁺ T cells further support CD8⁺ T‐cell function and establish long‐term immune memory through the secretion of IL‐21 and IL‐2 [242]. Additionally, cDC1s secrete CXCL9 and CXCL10, recruiting more effector cells, such as NK cells, into the tumor tissue [104, 105]. Research has indicated that a higher abundance of cDC1s in tumors generally predicts a better prognosis and is positively correlated with CD8⁺ T‐cell infiltration [243]. cDC2s also play a key role in tumor immunity. cDC2s can robustly activate CD4⁺ T cells and, under specific conditions, directly stimulate CD8⁺ T cells, thereby compensating for a cDC1 deficiency [244]. Research has shown that cDC2s express the IFN‐stimulating gene ISG, which can activate CD8^+^ T cells and mediate antitumor immunity in the absence of cDC1s [245]. pDCs play dual roles: they can exert antitumor effects by activating NK cells via TNF but are frequently co‐opted by the TME to adopt tolerogenic phenotypes, thereby facilitating immune evasion [246, 247]. pDCs contribute to an immunosuppressive TME through coinhibitory molecules (e.g., IDO, ICOS‐L, and PD‐L1) and Treg cell induction [243, 248]. Their presence is associated with a poor prognosis for various cancers, including breast, ovarian, head and neck squamous cell, and colon carcinomas [248]. In hepatocellular carcinoma (HCC), high pDC infiltration correlates with greater vascular invasion, an advanced stage, higher recurrence rates, and shorter survival [248]. Moreover, the TME suppresses DC maturation, antigen‐presenting capacity, and proinflammatory cytokine expression through factors such as IL‐6, IL‐10, GM‐CSF, TGF‐β, PGE2, VEGF, and ROS, driving DCs toward tolerogenic or immunosuppressive phenotypes [243].

Given the central role of DCs in tumor immunity, DC‐based vaccines have shown promising therapeutic potential. By activating tumor‐specific T cells, DC vaccines can suppress tumor growth and prevent recurrence via immunological memory. Two major approaches are used for DC vaccine preparation: (1) monocytes isolated from peripheral blood are differentiated into DCs with GM‐CSF and IL‐4, followed by maturation with TNF‐α; or (2) CD34⁺ progenitors are mobilized from bone marrow and cultured with GM‐CSF, Flt3L, and TNF‐α [103, 249]. These mature DCs can internalize and process various antigens (nucleic acids, peptides, proteins, and whole cells) for presentation to activate the immune system, thereby eliciting antitumor immune responses for prevention or treatment [250, 251]. Antigen‐loaded DC vaccines also promote IFN‐γ secretion by T cells, enhancing tumor cell death [249]. Current tumor DC vaccines can be divided into peptide‐based and genetic‐based types. Peptide‐based DC vaccines include those pulsed with tumor‐specific epitopes that are generated by fusing tumor cells with DCs, sensitized with tumor nucleic acids, or utilizing exosome vectors [250]. Genetic DC vaccines involve engineering DCs by transfecting them with tumor DNA, RNA, or cytokine genes to express tumor‐associated antigens or immune‐enhancing molecules or to downregulate negative regulators of DC function, thereby strengthening the three signals required for CD4⁺ and CD8⁺ T‐cell activation [252, 253]. This method enhances their antigen presentation and T‐cell activation capacities, optimizes costimulatory signals, alters the TME, and recruits other immune cells (NK cells and B cells) to induce more effective specific antitumor immunity [252, 253]. Both peptide‐ and gene‐based DC vaccines have shown antitumor efficacy in breast cancer, with some progressing to clinical trials [249]. For example, HPV16E7‐pulsed DC exosomes have been shown to suppress macrophage migration and inflammation, induce M2 polarization, and inhibit cervical cancer progression [254]. Sipuleucel‐T, an ex vivo‐generated DC vaccine, has been shown to prolong the overall survival of patients [255, 256]. However, its clinical activity as a monotherapy is modest, while increased efficacy is observed when it is combined with other immunomodulatory therapies [255, 256].

The limited efficacy of DC vaccine monotherapy has spurred the exploration of combination strategies, such as conventional chemotherapy, immune adjuvants (e.g., TLR agonists), mono‐/bispecific antibodies, and immune checkpoint‐targeting therapies. Combining DC vaccines with hypomethylating agents significantly enhances immune responses and treatment outcomes in patients with acute myeloid leukemia [257]. A study by Rob et al. [258] using an autologous active cellular immunotherapy composed of ovarian cancer DCs combined with carboplatin and paclitaxel for epithelial ovarian cancer showed significantly improved progression‐free survival (PFS) in the combination group, with a 61% reduction in the risk of progression and a 60% reduction in the risk of death compared with chemotherapy alone. Similarly, a study combining DC vaccines with the TLR agonist poly‐ICLC or resiquimod for malignant glioma demonstrated that poly‐ICLC amplified IFN‐induced gene expression in monocytes and T lymphocytes, prolonging patient survival and delaying disease progression [259]. Furthermore, tumors often suppress immune responses via mechanisms such as the PD‐1/PD‐L1 and CTLA‐4 pathways [260, 261]. Blocking these pathways can reverse T‐cell exhaustion and enhance DC vaccine‐induced immune responses [46, 260, 261]. Combining cDC1‐based vaccines with anti‐PD‐1 antibodies has shown promise, but compared with conventional ICIs, bispecific DC–T‐cell engagers may further improve intratumoral DC–T‐cell cross‐talk [262]. The combination of DC vaccines with anti‐PD‐1 immune checkpoint inhibitors increases T‐cell activity and antitumor effects on various cancer models, including HCC, glioblastoma, lung cancer, and gastric cancer models [263, 264, 265, 266]. Similarly, combining DC vaccines with CTLA‐4 inhibitors has improved the efficacy in both breast cancer and colon cancer [267, 268].

The integration of next‐generation sequencing and bioinformatics tools is revolutionizing cancer immunotherapy by enabling the identification of tumor‐specific neoantigens for designing personalized DC vaccines [269]. Li et al. [270] combined next‐generation sequencing with bioinformatics algorithms to establish and optimize a DNA vaccine platform targeting multiple neoantigens in metastatic pancreatic neuroendocrine tumors. The optimized multiepitope neoantigen DNA vaccine effectively induced antitumor immune responses, and the immunogenicity was significantly increased by the addition of mutant tags [270]. Personalized neoantigen‐based vaccines have also been evaluated in melanoma, glioblastoma, and pancreatic cancer [271, 272, 273].

Nanotechnology is also emerging as a powerful tool to enhance DC‐mediated antitumor immunity. Targeting DCs using nanoparticles (NPs) has emerged as a highly promising research direction in cancer immunotherapy. NP‐based targeting offers multiple advantages. (1) Intrinsic adjuvant activity (e.g., gold nanorods and carbon black NPs) can directly activate DC maturation, synergistically enhancing antigen immunogenicity to increase humoral/cellular immunity [274]. (2) Optimized physicochemical properties enable the precise delivery of antigens to mature DCs in lymphoid organs, avoiding immune tolerance induced by iDCs [274, 275]. (3) The NP structure can shield peptides from proteolytic degradation, ensuring antigen stability [276, 277]. (4) NPs serve as versatile platforms for codelivering antigens with adjuvants (e.g., TLR agonists) or siRNAs to silence immunosuppressive genes, synergistically increasing the potency of weakly immunogenic antigens [274, 276, 277]. (5) Surface modifications enable the controlled release of therapeutic components within DCs, ensuring prolonged activation [274, 278]. Breakthroughs in nanocarrier design focus on size effects and receptor targeting: small particles (25–40 nm) can penetrate tissue barriers directly to lymph nodes, whereas larger particles (>100 nm) rely on DC cellular uptake and transport [279]. Studies using chitosan‐lactate NPs loaded with an siRNA to suppress CTLA‐4 expression on tumor‐infiltrating T cells have shown promising synergistic antitumor effects [280]. In another study, rapamycin‐loaded poly(lactic‐co‐glycolic acid) NPs modified with DC membranes crossed the blood–brain barrier, remodeled the TME, and activated antitumor immunity, significantly suppressing glioma growth and inducing glial cell differentiation [281].

### 5.2 DCs and Diabetes

#### 5.2.1 Type 1 Diabetes Mellitus

T1DM is a T‐cell‐mediated autoimmune disorder characterized by immune attack against insulin‐producing pancreatic β‐cells, leading to their progressive dysfunction and loss [282, 283]. β‐Cell antigen‐specific CD8⁺ and CD4⁺ T cells are implicated in promoting the destruction of these insulin‐producing cells. Studies have indicated that DCs and macrophages are among the first immune cells to infiltrate islets in mouse models of T1DM [284, 285]. In the early stages of disease, innate immune cells (DCs and macrophages) infiltrate the islets, where they recognize and capture self‐antigens released from β‐cells, such as insulin, glutamate decarboxylase (GAD65), insulinoma‐associated protein 2 (IA‐2), and zinc transporter 8 (ZnT8) [286]. These antigens are processed by DCs and presented in pancreatic draining lymph nodes, where they activate naïve T cells. The resulting islet‐specific T cells then migrate into pancreatic islets: CD8⁺ T cells directly mediate the cytotoxic killing of β cells by recognizing β‐cell surface antigens, while CD4⁺ T cells differentiate into effector subsets such as Th1, Th2, and Th17 cells, which produce proinflammatory cytokines (e.g., TNF‐α, IFN‐γ, and IL‐2) [287]. These cytokines stimulate CD8⁺ T cells and macrophages, amplifying local inflammation and accelerating β‐cell destruction to thereby drive T1DM progression [287]. Moreover, synergistic interactions between T cells, NK cells, and B cells further exacerbate β‐cell damage [288]. As mentioned previously, DCs closely influence all these cell types, further underscoring their role in T1DM pathogenesis. Moreover, IFN‐secreting pDCs accumulate in the pancreas, triggering diabetes‐associated T‐cell responses and promoting T1DM progression in nonobese diabetic mice [289]. Specifically, locally released IFN‐I induces aberrantly high expression of human leukocyte antigen class I (HLA‐I) on islet cells, facilitating T‐cell activation and ultimately leading to β‐cell destruction [290]. Parackova et al. [291] found that in T1DM patients, moDCs respond vigorously to neutrophil extracellular trap (NET) fragments, as evidenced by significantly increased expression of maturation markers, increased production of inflammatory cytokines, and an improved capacity to induce the production of IFN‐γ‐secreting T lymphocytes. Furthermore, NET‐stimulated moDCs from T1DM patients shifted toward anaerobic glycolysis, further corroborating their proinflammatory state [291]. On the other hand, DCs can play a dual role in T1DM pathogenesis and progression: they can promote inflammatory T‐cell responses but also mediate immune tolerance under appropriate conditions, making them key immunoregulators influencing the disease course. For instance, as highlighted in a review by Giannoukakis, tolDCs can block autoimmune attack through multilayered immunoregulatory mechanisms, helping to preserve residual β‐cell function in patients with newly diagnosed T1DM [292].

Given the critical role of DCs in T1DM pathogenesis, DC‐based cellular therapies hold great promise. One of the most advanced strategies is the generation or induction of tolDCs, either ex vivo or in vivo. Ex vivo, monocytes can be differentiated into iDCs in the presence of growth factors such as GM‐CSF and cytokines such as IL‐4 [293]. This differentiation process is combined with immunomodulators, including anti‐inflammatory cytokines (e.g., IL‐10 and/or TGF‐β) or specific pharmacological agents (e.g., dexamethasone, rapamycin, and vitamin D3), to impart immunotolerant properties [293]. In vivo, the number of functional tolDCs can be increased using antisense oligonucleotides, liposomes, TLR ligands, islet peptides, or cytokines [293]. Torres‐Aguilar et al. [294] generated moDCs using GM‐CSF and IL‐4 and converted them into alternatively activated DCs by adding IL‐10 and TGF‐β. These DCs induced insulin‐specific tolerance in effector/memory CD4⁺ T cells from T1DM patients, characterized by reduced T‐cell proliferation, decreased IL‐2 and IFN‐γ expression, and increased IL‐10 secretion. Similarly, Ferreira et al. [295] demonstrated that tolDCs treated with 1,25‐dihydroxyvitamin D3 [1,25(OH)_2_D_3_] acquire a tolerogenic phenotype in diabetes‐prone mice, suppress T‐cell activation, expand Treg cells, and increase IL‐10 production. Further studies showed that 1,25(OH)_2_D_3_‐treated tolDCs exhibited an excellent migratory capacity, effectively homing to the liver and pancreas, and efficiently suppressed the proliferation of donor autoreactive T cells in pancreatic draining lymph nodes.

DC‐based vaccination has also been explored in T1DM patients. Clinical studies have shown that autologous DC therapy increases beneficial B220⁺ CD11c^−^ B‐cell populations and exhibits safety and tolerability in both natural and induced tolerogenic states [296]. In one trial, intradermal injection of insulin peptide‐pulsed tolDCs stabilized β‐cell function and glycemic control over six months, with stable HbA1c levels and unchanged insulin requirements [297]. Another study demonstrated that the tolDC vaccine significantly and persistently suppressed the original autoimmune response to the vaccine peptides within three years after treatment while temporarily reducing the CD4^+^ and CD8^+^ T‐cell responses to other pancreatic islet autoantigens [23].

Several emerging technologies combined with DC therapy show promise for treating T1DM. First, genetic engineering techniques can be used to “reprogram” DCs to increase their tolerance and Treg cell‐inducing capacity for T1DM treatment [298, 299, 300]. Common gene therapy strategies include the introduction of inhibitory costimulatory molecules (e.g., PD‐L1 and CTLA‐4) or factors that enhance immune tolerance (e.g., IL‐10 and TGF‐β) [301, 302]. For example, genetically modifying DCs to overexpress IL‐10 can significantly suppress overactive T‐cell responses and mitigate immune attack on pancreatic β‐cells [303]. Second, stem cell technology is being applied. Studies have shown that human periodontal ligament stem cells (PDLSCs) can significantly inhibit the expression of immunogenic markers (including CD80, CD83, CD86, CD40, CD1a, CD209, and HLA‐DR) on mature DCs derived from T1DM patients while increasing the secretion of IL‐6 and TGF‐β, exerting substantial immunosuppressive effects [304]. These findings suggest that PDLSCs might alleviate immune destruction in patients with diabetes by modulating DC function. Research has also revealed that extracellular vesicles derived from mesenchymal stem cells can improve immune tolerance in patients with T1DM through similar mechanisms, suppress islet inflammation, and promote anti‐inflammatory responses [304]. Embryonic stem cell‐derived DCs were shown to almost completely suppress diabetes development in prediabetic nonobese diabetic mice [305]. Furthermore, combining nanotechnology with DC therapy holds potential. Lewis et al. [306] used PLGA NPs to modulate DC chemokines and recruit tolDCs, successfully increasing the number of Treg cells and reversing hyperglycemia. Engman et al. [307] found that nontargeting tolerogenic NPs could deliver autoantigens to the pancreas via the draining lymph nodes, inducing the production of Foxp3⁺ Treg cells and further promoting the establishment of immune tolerance.

#### 5.2.2 Type 2 Diabetes Mellitus

Type 2 diabetes mellitus (T2DM) is a chronic metabolic disorder characterized by insulin resistance (IR) and β‐cell dysfunction, and its pathogenesis is closely linked to chronic low‐grade inflammation. DCs contribute to T2DM by promoting IR and β‐cell dysfunction through their role in this inflammatory state, reducing insulin secretion and thus acting as significant drivers of the disease. Adipose tissue DCs accumulate around lymphoid aggregates, where they monitor antigen trafficking from AT to lymph nodes, regulate immune responses, and mediate AT inflammation [308]. Studies have shown that the number of DCs is increased in the AT of high‐fat diet‐fed mice, secrete higher levels of proinflammatory cytokines, and are more potent at inducing the activation of Th1 and Th17 cells associated with AT inflammation [309, 310, 311]. IL‐6 released by DCs can promote the polarization of macrophages toward the M1 phenotype, thereby exacerbating inflammation in adipose tissue [312]. Depleting DCs reduces inflammatory cytokine levels, suppresses the obesity‐induced increase in the number of CD4⁺ T cells in visceral AT, and alleviates AT inflammation [309, 310, 313]. Adipokines (e.g., adiponectin, leptin, visfatin, and chemerin) can also influence DCs through various mechanisms, amplifying local inflammation, inhibiting insulin signaling, and leading to IR [314, 315, 316]. In addition to AT, hepatic and skeletal muscle DCs play distinct roles in T2DM progression. The liver has a unique immune microenvironment enriched with sinusoidal endothelial cells, Kupffer cells (KCs), and DCs. Hepatic DCs sense portal vein‐derived PAMPs/DAMPs, such as LPS and free fatty acids (e.g., palmitic acid and oleic acid), leading to their activation [317]. Activated hepatic DCs produce IL‐6, TNF‐α, and IL‐23, recruiting and activating Th1/Th17 cells to exacerbate local inflammation [317]. Hepatic DC activation is also a key driver of NAFLD progression to NASH and is closely correlated with hepatic IR and systemic metabolic dysregulation [318]. DC recruitment to the liver depends on KCs, and interactions between DCs and KCs amplify the inflammatory milieu, impairing insulin receptor substrate phosphorylation and disrupting insulin signaling [319]. Similarly, the number of skeletal muscle DCs increases in obese individuals, and these cells secrete proinflammatory mediators and interfere with insulin sensitivity. DC‐activated T cells release IFN‐γ and other cytokines, forming a “metabolic stress–DC activation–muscle IR” axis that reduces glucose uptake in muscle. Pancreatic DCs also contribute to this axis by secreting proinflammatory cytokines and promoting T‐cell infiltration and activation, further damaging β cells [320, 321].

Unlike conventional interventions targeting glucose levels or insulin sensitivity, strategies directed at DC‐driven inflammation have the potential to address T2DM at its root by restoring the metabolic balance. In recent years, various anti‐inflammatory strategies based on DC modulation have emerged. For example, Sun et al. [322] reported that acupuncture inhibited DC activation, leading to reduced numbers of IL‐17‐producing T cells and Th1 cells, thereby alleviating psoriasis‐related inflammation. Ginwala et al. [323] demonstrated that apigenin reprograms DC‐mediated T‐cell responses from proinflammatory Th1/Th17 cells toward Treg cells, downregulating T‐bet, IFN‐γ, and IL‐17 while increasing IL‐10, TGF‐β, and FoxP3 expression and thus alleviating neuroinflammation. Xiang et al. [324] reported that kinsenoside suppresses DC maturation, increases PD‐L1 expression, and reduces IL‐12 secretion, thereby blocking CD8⁺ T‐cell and hepatic stellate cell activation and mitigating liver injury and fibrotic inflammation. Toshimitsu et al. [325] found that *Lactobacillus plantarum* OLL2712 induced IL‐10 production in murine DCs and macrophages, suppressed chronic inflammation and improved metabolic disorders, thus protecting against T2DM. Collectively, these studies confirm the efficacy of DC‐targeted inflammatory modulation, providing new therapeutic avenues for alleviating chronic low‐grade inflammation and metabolic dysregulation in T2DM patients.

Furthermore, immunomodulatory therapies have been shown to be effective treatments for T2DM [326]. Although T2DM is traditionally classified as a nonautoimmune disorder, immune dysregulation plays an important role in its pathogenesis [327, 328]. A chronic immune imbalance drives persistent inflammation, which contributes to IR and β‐cell dysfunction [326, 329]. Therefore, the central role of DCs in immune responses makes them promising new targets for T2DM treatment. Studies have shown that cDCs can promote IL‐10 production via β‐catenin signaling, thereby maintaining an anti‐inflammatory environment [330]. However, in individuals with obesity, β‐catenin signaling in cDCs is weakened, exacerbating local inflammation. Macdougall et al. [331] showed that constitutively activating β‐catenin signaling in cDCs could reverse tissue inflammation and improve glucose homeostasis by promoting the hyperplasia of pancreatic islets, thereby increasing insulin release and improving the immune environment. Additionally, sitagliptin, an oral medication widely used to treat T2DM, has been identified as a drug that targets DCs. It increases the efficacy of cancer immunotherapy by promoting the DC‐mediated priming of antigen‐specific T cells, suggesting that it might also target DCs to ameliorate immune dysregulation in individuals with T2DM [332]. Thus, DC‐based immunomodulatory strategies are potentially worth exploring in future T2DM research.

### 5.3 DCs and Infectious Diseases

Infectious diseases, caused by pathogenic microorganisms such as bacteria, viruses, and fungi, are characterized by high transmissibility, variability, and immune evasion capabilities [333]. These pathogens often achieve persistent infection by targeting key nodes of the host immune system, preventing the host from clearing them effectively. In this context, DCs serve as sentinel cells of the immune system and key initiators of adaptive immunity, with their functional state critically shaping infection outcomes. DCs recognize PAMPs via PRRs, including TLRs, CLRs, RLRs, and cytosolic DNA sensors such as cGAS [334, 335]. For instance, viruses with ssRNA genomes (e.g., SARS‐CoV‐2, IAV, and HIV) are recognized by TLR7, RIG‐I, and MDA5, whereas viruses with DNA genomes (e.g., HHV) are recognized by TLR9, cGAS, and IFI16. CLRs (e.g., DC‐SIGN) can recognize glycoprotein structures on various viruses [336]. Furthermore, abnormal molecules produced during viral infection, such as replication intermediates, released mitochondrial DNA, or double‐stranded RNA, can activate the NLRP3 inflammasome or signaling pathways such as TLR3 and cGAS–STING, further strengthening DC activation [336]. This broad pathogen‐sensing capacity makes DCs indispensable “commanders” of antiviral immunity. However, pathogens, especially viruses, have evolved sophisticated strategies to interfere with and inhibit DC function to achieve immune evasion and persistent infection. Notably, despite vast differences in the taxonomic status, genomic structure, tropism, life cycle, and epidemiological features (e.g., SARS‐CoV‐2, IAV, HIV, and HHV), viruses consistently target DCs as a key strategy for immune escape [337]. These strategies share the following significant commonalities: (1) suppressing antigen presentation and costimulation by downregulating MHC expression and impairing antigen processing; (2) impairing DC migration to draining lymph nodes, thus blocking the initiation of adaptive immunity; (3) exploiting DCs as viral carriers, such as in HIV and HHV, which bind DC‐SIGN to disseminate systemically; and (4) interfering with critical signaling pathways, including TLR, RLR, and cGAS–STING signaling, thereby suppressing type I IFN and proinflammatory cytokine production. The convergent evolution of DC‐targeting immune evasion underscores the central role of DCs in anti‐infective immunity and highlights their potential as therapeutic targets.

Therefore, DC‐based vaccines for infectious diseases have been extensively studied. Ex vivo peptide‐loaded DC vaccines are typically used to treat infectious diseases [25]. Autologous moDCs loaded with inactivated autologous virus (e.g., via Aldrithiol‐2 or heat inactivation) showed clinical potential for treating chronic HIV‐1 infection: the median plasma viral load decreased by 80%, with >90% suppression achieved in some patients, which correlated with the expansion of HIV‐specific CD4⁺ Th1 cells (IL‐2/IFN‐γ‐secreting) and perforin⁺ CD8⁺ effector T cells [338, 339]. Encke et al. [340] reported that DC vaccines loaded with HCV‐specific antigens enhanced both humoral and cellular immune responses in HCV‐infected mice. Similarly, numerous studies have shown good efficacy of ex vivo peptide‐loaded DC vaccines against viral infections (e.g., influenza and [341] herpes simplex virus [342, 343]), fungal infections (e.g., *Candida albicans* [344] or *Cryptococcus gattii* [345]), and parasitic diseases (e.g., visceral leishmaniasis [346, 347]).

In vivo, DC‐targeted vaccines represent another promising modality [25]. DC‐targeting vaccines can be achieved through nanocarrier design and genetic engineering. For example, the DermaVir nanovaccine, which is based on polyethyleneimine–mannose, targets the DC‐SIGN receptor and significantly increases CD4⁺/CD8⁺ T‐cell responses and cytokine secretion in HIV clinical trials [279]. Compared with free peptides, gold NP–mannose–HIV peptide conjugates induced markedly stronger T‐cell activation [279, 348]. β‐Glucan particles targeted dectin‐1 on DCs, eliciting Th1/Th17 polarization and the production of protective antibodies in fungal vaccine models [349]. Innovative delivery routes further improved the outcomes: transdermal delivery of Man‐PEI *nanovaccines* targeted DC‐rich skin, intravaginal delivery of Sap2‐virosome vaccines eliminated *Candida* infection [349], and intranasal administration of poly‐ICLC with CpG–ODN adjuvants during COVID‐19 induced mucosal sIgA and tissue‐resident T‐cell production, providing dual‐layered respiratory protection [348]. Although ex vivo DC vaccines (e.g., for HIV and COVID‐19) are safe and bypass in vivo targeting barriers, their complexity and cost remain major obstacles [350]. Future efforts need to focus on developing low‐cost in vivo targeting carriers, exploring the synergistic effects of TLR/STING agonists, and optimizing mucosal delivery to induce tissue‐resident immunity. Notably, DC vaccines can reconstitute functional T‐cell responses even in immunocompromised populations (e.g., HIV patients), highlighting their unique value as pan‐infectious disease treatment platforms [349, 350]. Genetic engineering further expands DC‐targeting vaccine strategies by fusing antigens with single‐chain variable fragments specific for DC receptors [25]. For example, engineered monoclonal antibodies targeting DEC‐205 effectively induced CD4⁺ and CD8⁺ T‐cell responses, conferring protection in models of *Leishmania major*, *Yersinia pestis*, recombinant vaccinia virus, and HIV‐1 [25]. Other receptors, such as DC immunoreceptor and CD40, have also been exploited for precise antigen delivery to DCs [351].

Combination therapies have shown synergistic efficacy. Optimized adjuvants are critical for DC activation. TLR agonists such as CpG–ODN and poly‐ICLC enhance vaccine efficacy by overcoming tolerance. DEC‐205 antibody‐modified NPs codelivering TLR3/7/8 agonists and antigens significantly increased DC activation [349, 352]. A novel “super TLR agonist” combining a modified TLR5 ligand with a SOCS1 inhibitor synergized with DC vaccines to induce sustained cytokine production and robust HCV‐specific immune responses [353]. Furthermore, combining DC activation strategies with antiviral drugs, neutralizing antibodies, or other immunomodulators can achieve synergistic effects.

### 5.4 DCs and Transplantation

As key sentinels of the immune system, DCs play dual roles in transplant immunity by both promoting rejection and inducing tolerance. Recent studies have shown that dynamic changes in peripheral blood myeloid DCs can predict the risk of acute graft‐versus‐host disease (aGVHD) following allogeneic HSC transplantation, as pretransplant myeloid DC proportions and counts are significantly positively correlated with the incidence and severity of aGVHD, whereas myeloid DCs during the engraftment phase exhibit a protective negative correlation [354]. Notably, their predictive sensitivity surpasses that of pDCs, indicating that myeloid DCs can serve as biomarkers for early intervention [354]. This predictive value arises from the precise immune microenvironment‐sensing capacity of DCs. During solid organ transplantation, DCs regulate the immune balance through phenotypic conversion. For example, in corneal transplantation models, migratory CD103⁺ DC1s exhibit a tolerogenic phenotype (high expression of IL‐10, PD‐L1, and αvβ8 integrin) in low‐inflammatory environments [355]. These DCs suppress the immunogenicity of CD11b⁺ DC2s, block Th1 cell activation via the PD‐L1/PD‐1 pathway, and promote Treg cell differentiation via the αvβ8–TGF‐β1 axis, thereby maintaining graft survival. Conversely, in a high‐inflammatory microenvironment, IFN‐γ drives CD103⁺ DC1s to adopt a proinflammatory phenotype (upregulating IL‐12/CD80/86 and downregulating regulatory molecules), enhancing the antigen presentation capacity of CD11b⁺ DC2s and blocking Th1 cell exhaustion to ultimately lead to rejection [355]. Notably, the adoptive transfer of ex vivo‐induced tolerogenic CD103⁺ DC1s can reshape immune homeostasis and significantly prolong graft survival, highlighting the direct therapeutic potential of agents targeting DC plasticity [355]. Moreover, the regulatory functions of DCs exhibit significant organ‐specific heterogeneity. In liver transplantation, cDCs display tolerogenic features under steady‐state conditions (low MHC‐II expression and high PD‐L1 expression), but ischemia‒reperfusion injury drives their maturation via the expression of inflammatory cytokines such as TNF‐α/IFN‐γ, upregulation of CCR7 expression, and subsequent migration to lymph nodes where they activate T cells [18]. Moreover, pDCs promote Treg cell differentiation via a high PD‐L1‐to‐CD80/86 ratio and IDO secretion, although they can convert to a proinflammatory phenotype upon TLR stimulation [18]. In contrast, kidney transplant rejection is mediated primarily by moDCs. Nonclassical monocytes differentiate into IL‐12⁺ DCs after transplantation, accelerating graft dysfunction by promoting effector T‐cell proliferation and survival [356]. These organ‐specific differences suggest that targeted DC therapies should be tailored to the local microenvironment.

Based on these mechanisms, three main DC‐based interventional strategies have been developed to counteract transplant rejection. First, immunosuppressive drugs can be used to reprogram DC phenotypes. Existing immunosuppressive agents can promote tolerance induction by directly modifying DC functions: the calcineurin inhibitor tacrolimus (FK506) promotes high expression of IL‐10/TGF‐β in DCs, significantly inhibiting CD4⁺ T‐cell proliferation [357]; the mTOR inhibitor rapamycin (RAPA) downregulates costimulatory molecules (CD80/86), upregulates the immune checkpoint PD‐L1 and immunoglobulin‐like transcription factors (ILT3/ILT4), and promotes Treg cell expansion [358]; and glucocorticoids, such as dexamethasone, delivered via NP formulations not only inhibit DC maturation but also induce tolDC phenotypes. This process induces a tolerogenic phenotype through the downregulation of MHC‐II/CD40/CD80 and proinflammatory cytokines (TNF‐α/IL‐1β), blocking T‐cell activation [359]. Second, precise gene engineering strategies to regulate DC functions are needed. Studies have shown that silencing the circular RNA circMAP2K2 in combination with the deSUMOylase SENP3 reduce the expression of MHC‐II/CD40/CD80 on DCs, weakening their ability to activate alloreactive T cells while promoting CD4⁺CD25⁺FOXP3⁺ Treg cell induction. This strategy has been shown to achieve donor‐specific immune suppression in mouse heart transplants [360]. Additionally, XBP1‐deficient BMDCs use the IRE1‐dependent pathway to degrade the TAPBP mRNA, reducing MHC‐I expression and inhibiting CD8⁺ T‐cell‐mediated cytotoxic rejection, with low‐dose cyclosporine A synergistically prolonging heart transplant survival [361]. Third, adoptive tolDC therapy is recommended. Significant breakthroughs in the translation of ex vivo‐induced tolDCs have been achieved. When a GM‐CSF/IL‐4 culture system supplemented with tolerance‐inducing agents (vitamin D3, rapamycin, and IL‐10) or gene editing is used, tolDCs with an MHC‐II^low^/CD80/86^low^/IL‐10^high^/TGF‐β^high^ phenotype can be generated [18, 362, 363]. The core mechanism involves inducing T‐cell anergy/apoptosis, expanding antigen‐specific Treg cells, and inhibiting memory T‐cell activation [18, 364]. Currently, two clinical trials involving liver transplant recipients (NCT03164265 and NCT04208919) are evaluating the safety and efficacy of the combination of donor‐derived tolDCs and conventional immunosuppressive agents. Nonhuman primate models have confirmed that this approach reduces inflammatory cell infiltration and extends graft survival [18].

### 5.5 DCs in Other Diseases

DCs also play important roles and have therapeutic potential in various nonmetabolic diseases, including autoimmune disorders (e.g., RA, multiple sclerosis [MS], and systemic lupus erythematosus [SLE]), neurodegenerative diseases (e.g., Alzheimer's disease [AD] and Parkinson's disease [PD]), cardiovascular diseases (e.g., atherosclerosis and myocardial infarction), allergic diseases (e.g., asthma, allergic rhinitis, and atopic dermatitis), and dermatological conditions (e.g., psoriasis and vitiligo).

In individuals with RA, DCs promote synovial inflammation and joint destruction through excessive T‐cell activation and proinflammatory cytokine secretion [365]. In individuals with MS, infiltrating DCs present myelin antigens within the CNS, directly driving neuroinflammation and demyelination [366]. In individuals with SLE, defective antigen presentation triggers autoreactive T and B‐cell activation, autoantibody production, and immune complex deposition, exacerbating tissue injury [367]. TolDCs have shown promising efficacy in experimental and early clinical studies of RA [368], MS [369], and SLE [370]. Studies indicate that tolerogenic DEC205^+^ DC vaccines loaded with autoantigens hold clinical potential for RA treatment, although their successful application in humans depends on a deeper understanding of the relevant autoantigens [371]. Furthermore, a study using antigen‐peptide‐loaded NPs to induce tolDCs in vivo showed significant therapeutic benefits in individuals with SLE [372].

In individuals with AD, impaired clearance of amyloid‐β (Aβ) by peripheral DCs and their senescence‐associated decrease in antigen presentation weaken systemic amyloid clearance. Concurrently, abnormal interactions between brain DC‐like cells and the pathological Tau protein may exacerbate the neuroinflammatory microenvironment [373]. A rationally designed DC‐targeted nanovaccine incorporating Aβ and rapamycin elicited the production of both anti‐Aβ antibodies and Aβ‐specific Treg cells in preclinical models, effectively clearing plaques, restoring cognition, and, crucially, avoiding detrimental Th1 responses associated with conventional vaccines [374]. Given the synergistic roles of Aβ and tau in AD pathology, DC vaccines may coordinate adaptive immune responses to multiple pathogenic proteins. By driving Th2‐biased and Treg cell responses, they can simultaneously alleviate the Aβ and tau burdens, reprogram microglia toward neuroprotective phenotypes, suppress neuroinflammation, enhance protein clearance, and support neuronal survival; thus, they represent an integrated strategy for immunoregulation and neuroprotection [373, 375]. In individuals with PD, DCs present α‐synuclein to T cells, triggering cytotoxic responses toward dopaminergic neurons while also facilitating the intercellular spread of misfolded α‐synuclein [376]. Immunomodulatory strategies targeting DCs show significant therapeutic potential: GM‐CSF‐induced tolerogenic BMDCs drive Treg cell differentiation via OX40L/Jagged‐1 signaling. Adoptive transfer of these BMDCs in an MPTP model significantly increased the splenic Treg cell frequency, suppressed microglial activation by 58%, and protected nigrostriatal dopaminergic neurons [377]. Notably, PD patients exhibit a deficiency of circulating PD‐L1⁺ tolDCs, which is correlated with systemic reductions in the numbers of Treg cells, Breg cells, and CD8 regulatory cells, suggesting that adoptive tolDC therapy may restore immune homeostasis and suppress neuroinflammation [378].

In atherosclerotic lesions, DCs activate T cells by recognizing lipid‐related antigens and secreting proinflammatory cytokines, exacerbating chronic vascular wall inflammation and promoting lipid deposition, plaque formation, and instability [379]. Recent studies have revealed that DC‐targeted immunotherapies, such as the induction of tolDCs, the development of oxLDL‐loaded DC vaccines, or the application of DC‐derived exosomes carrying miR‐203‐3p, can promote Treg cell expansion, suppress inflammatory signaling pathways, and inhibit DC maturation, thereby reversing the proinflammatory microenvironment and stabilizing atherosclerotic plaques [380, 381, 382, 383, 384]. In individuals with myocardial infarction, DCs present cardiac antigens to T cells, sustaining inflammatory responses and worsening injury, which drives left ventricular remodeling and heart failure [385]. In contrast, tolDCs preconditioned with cardiac lysates expand Treg cells, polarize macrophages toward the reparative M2 phenotype, and significantly improve angiogenesis and cardiac function (left ventricular ejection fraction increased by 37%) [386].

In individuals with asthma, DCs capture inhaled allergens and present them to T cells, initiating Th2 responses that promote IgE production and airway inflammation [387]. Airway epithelial cells regulate DC activation and migration via secreted mediators, sustaining allergic responses [387]. In individuals with allergic rhinitis, mature DCs interact with CD4⁺ Th2 cells, increasing IgE production and eosinophilic inflammation in nasal tissue [388]. In individuals with atopic dermatitis, DCs drive Th2‐skewed responses and secrete chemokines that recruit Th2 cells to the skin, amplifying local inflammation [389]. Therapeutic strategies are emerging: gene‐engineered DCs overexpressing IL‐38 induce the production of Treg cells and suppress Th2‐mediated inflammation [390]; targeted delivery systems such as P‐D2‐EV nanovesicles can reprogram DC metabolism via the Fut1/ICAM1/p38 MAPK axis, inhibiting ILC2 activation [391]; and tissue‐resident DC subsets present during remission may serve as targets for prolonging tolerance and preventing relapse [392].

In individuals with psoriasis, DCs are major sources of IL‐23, IL‐12, and TNF‐α and activate Th17/Th1 cells to produce IL‐17 and IL‐22, which drive keratinocyte hyperproliferation and chronic inflammation [393]. Notably, aberrant activation of the cGAS–STING signaling pathway in DCs amplifies IL‐17A production by Th17/γδ T cells via increased IL‐23 secretion. Targeted inhibition of STING (e.g., by the specific knockout of the Sting gene in DCs or treatment with the inhibitor C‐176) significantly reduces DC activation and downstream IL‐17A generation, markedly alleviating psoriatic inflammation and exerting synergistic effects with anti‐IL‐17A therapy [322]. DCs sense oxidative stress signals (e.g., HSP70) and secrete IL‐12, IL‐17A, and TNF‐α, inducing Th1/Th17 responses and promoting CD8⁺ T‐cell infiltration in the skin [394]. Concurrently, a marked reduction in immunomodulatory, anti‐inflammatory DC subsets occurs, creating a proinflammatory‐dominant immune environment that drives melanocyte destruction and depigmentation [394]. Consequently, targeting aberrant DC activation has become a core focus of novel therapeutic strategies. Preclinical studies have confirmed that gene therapy delivering mutant HSP70i can block DC activation and the expression of maturation markers (CD80/CD86/HLA‐DR), effectively suppressing T‐cell‐mediated autoimmune responses and achieving sustained repigmentation in animal models [395, 396, 397].

### 5.6 Preclinical and Clinical Studies of DC‐Based Therapies

DCs have been extensively investigated for the treatment of various diseases, with applications extending from fundamental mechanistic studies to broad preclinical exploration and clinical translational research (Table 2). Preclinical research encompasses a variety of disease models, including allergic rhinitis, asthma, SLE, RA, heart failure, PD, AD, tuberculosis, acute kidney injury, systemic *Candida albicans* infection, avian influenza (H9N2), EAE, myocardial infarction, xenotransplant rejection, and dilated cardiomyopathy models. Clinical research extensively covers numerous disease areas. In oncology, it primarily involves solid tumors such as prostate cancer, non‐small cell lung cancer (NSCLC), HCC, melanoma, pancreatic cancer, breast cancer, ovarian cancer, glioblastoma, gastrointestinal stromal tumors, small cell lung cancer (SCLC), head and neck cancer, and mesothelioma. In the field of infectious diseases, clinical research has been conducted on HIV, HCV, and COVID‐19. Significant progress has also been made in exploring treatments for autoimmune diseases, including T1DM, MS, neuromyelitis optica spectrum disorder (NMOSD), SLE, and RA.

**TABLE 2:** Preclinical and clinical studies based on dendritic cells.

Study type	Experiment type	DC‐based interventions	Experimental subjects	Experimental results	Possible mechanism	Citations
Clinical trial (NCT03387553)	Phase I clinical trial	Type 1 DC vaccines targeting HER2 (HER2‐DC1)	Breast cancer patients	The administration of neoadjuvant DC1 vaccines is safe and feasible when given prior to neoadjuvant chemotherapy. The HER2 DC1 vaccine caused a significant influx of T cell infiltrates into treated tumors.	Reverse tumor mediated immunosuppression, re‐establish recognition of HER2 epitopes, and lead to the regression of early breast cancers when injected into draining axillary lymph nodes.	[398]
Clinical trial (NCT03113019)	Phase I/II clinical trial	Autologous DCs and activated cytotoxic T‑cells as combination therapy	Breast cancer patients	Improved 3‐year RFS; decreased peripheral blood Treg cell and MDSC; enhanced cytotoxicity against MCF‐7 cells	DCs loaded with tumor antigens activate cytotoxic T cells; polarize Th1 immune responses; and reduce immunosuppressive cell populations.	[399]
Clinical trial (NCT03450044)	Phase I/II clinical trial	Autologous DC combined with doxorubicin and cyclophosphamide	Patients with invasive ductal carcinoma of the breast	Restore T cell Zap70/AKT/mTOR phosphorylation function; increase peripheral and tumor‐shared TCR clones	DCs enhance tumor antigen cross‐presentation and T cell activation by leveraging chemotherapy‐induced immunogenic cell death.	[400]
Clinical trials (NCT02107937)	Phase II clinical trial	DCVAC/OvCa (autologous DC loaded with antigens from heterologous ovarian cancer cell lines)	Patients with epithelial ovarian cancer	Sequential administration significantly improved PFS (HR = 0.39) and had a good safety profile.	Sequential DC vaccination after chemotherapy may induce more durable antitumor immune responses.	[258]
Clinical trials (NCT02111941)	Phase I clinical trial	Autologous DC loaded with FRα peptide were induced to generate Th17 type by p38 inhibitor + IL‐15	Patients with stage IIIC–IV ovarian cancer	The vaccine is safe, inducing antigen‐specific T cell and antibody responses, and 39% of patients have long‐term no recurrence.	Induces Th17/Th1 cell and antibody responses, enhancing antitumor immunity and ADCC effects	[401]
Clinical trials (NR)	Phase I clinical trial	DC vaccine loaded with autologous tumor lysate	Patients with malignant glioma	Combination therapy is safe, with a low immune response rate (25%) and survival comparable to historical controls.	Tumor lysates provide multiantigen stimulation, but local chemotherapy may suppress immune responses.	[402]
Clinical trials (EudraCT: 2011‐001690‐62)	Phase II clinical trial	DC vaccine loaded with HepG2 cell lysate + opsonizing cyclophosphamide	Patients with intermediate‐stage HCC	According to RECIST 1.1 criteria, the median PFS in the DC vaccine group was 18.6 months, and that in the TACE + cyclophosphamide group was 10.4 months. The DC vaccine group also showed enhanced immune responses and AFP‐specific T cell responses.	Cyclophosphamide eliminates Treg cells, TACE releases antigens, and DC vaccine activates AFP‐specific T cells.	[403]
Clinical trial (KCT0000427)	Phase I/IIa clinical trial	DC vaccine pulsed with tumor‐associated antigens (AFP, GPC‐3, and MAGE‐1)	Patients with HCC who had no residual tumor postprimary treatment	Nine out of 12 patients were recurrence‐free up to 24 weeks after vaccination. The median time to progression was 36.6 months for the vaccinated group.	Activation of TAA‐specific T cells, inducing tumor‐specific immunity. The DCs were used to enhance T cell proliferation and activation of CTLs. The use of TLR‐7 agonist (imiquimod) facilitated DC migration to regional lymph nodes.	[404]
Clinical trial (NR)	Phase I clinical trial	DC vaccine loaded with autologous tumor stem cell lysate	Hepatitis B‐positive HCC patients	There was no significant liver toxicity or exacerbation of hepatitis B, and no obvious adverse reactions to DC vaccine treatment; local injection reactions were mild.	DCs present tumor stem cell antigens, activate antitumor immunity, and do not trigger the exacerbation of viral hepatitis.	[405]
Clinical trial (NCT02689644)	Phase I clinical trial	Intratumoral administration of inflammatory allogeneic DCs (ilixadencel)	Patients with advanced gastrointestinal stromal tumors patients	Four patients experienced tumor progression within 3 months; two patients (on second‐ and third‐line tyrosine kinase inhibitors, respectively) showed stable disease and partial responses at 3 and 6 months, respectively. The median PFS was 4 months.	Ilixadencel recruits NK cells, pre‐DCs, and T cells to the tumor microenvironment by secreting chemokines, promoting the cross‐presentation and maturation of endogenous DCs, and subsequently activating tumor‐specific T cells to induce a systemic antitumor immune response.	[406]
Clinical trial (NR)	NR	DC and CIK cells bioimmunotherapy	Advanced NSCLC patients	DC–CIK biological immunotherapy for advanced NSCLC has clinical efficacy. It can improve the immune function of patients, prolong their survival time within 2 years, and has certain safety.	DC with CIK cells enhances cell proliferation and killing activity, improving the specificity of tumor cell destruction. This helps eliminate microtumors and disseminated tumor cells, delaying or preventing tumor metastasis and recurrence.	[407]
Clinical trial (NCT02470468)	Phase I/II clinical trial	Autologous DC vaccine combined with carboplatin and paclitaxel chemotherapy	Patients with stage IV NSCLC	The median OS of the combination therapy group was prolonged by 3.7 months (15.5 vs. 11.8 months), with good safety.	Chemotherapy induces immunogenic cell death, enhances DC antigen presentation, and reverses the immunosuppressive microenvironment.	[408]
Clinical trial (NCT02122861)	Phase I clinical trial	LV305 (lentiviral vector targeting DCs), injected intradermally, expressing NY‐ESO‐1 antigen	Patients with NY‐ESO‐1‐positive advanced solid tumors	Good safety, induced T cell responses in 57% of sarcoma patients, and one synovial sarcoma patient achieved a partial response lasting >36 months	LV305 transfected DCs in vivo to express NY‐ESO‐1 and activate antigen‐specific CD4+/CD8+ T cell responses.	[409]
Clinical trial (NCT00617409)	Phase II clinical trial	Ad.p53‐DC (autologous DC vaccine transduced with wild‐type TP53 by adenovirus), intradermal injection	Patients with recurrent extensive‐stage SCLC	The vaccine is safe and induces immune responses in 20–40% of patients, but it does not significantly improve the response rate to second‐line chemotherapy (ORR = 23.8%).	Activation of tumor‐specific CD8+ T cells by p53 antigen combined with α‐GalCer may reduce MDSCs suppression.	[410]
Clinical trial (NCT01433172)	Phase I/II clinical trial	GM.CD40L vaccine (expressing GM‐CSF and CD40L), with or without CCL21	Patients with advanced lung adenocarcinoma	No significant difference in OS between GM.CD40L and GM.CD40L.CCL21 (9.3 vs. 9.5 months). 15.2% of GM.CD40L patients had 6‐month PFS.	CCL21 activates DCs through GM‐CSF and CD40L, recruits T cells, and enhances antitumor immune responses.	[411]
Clinical trial (NR)	Phase III clinical trial	Adoptive infusion of autologous regional lymph node‐derived activated cytotoxic T cells and DCs (AKT–DCs)	Patients with postoperative NSCLC (stages IB–IV)	The OS rate in the immunotherapy group was significantly improved compared with the no‐treatment group, with 2‐year and 5‐year OS rates of 96 and 69.4%, respectively.	Cytotoxic T cells directly kill circulating tumor cells, DCs present antigens, and enhance antitumor immunity.	[412]
Clinical trial (UMIN000010386)	Phase II clinical trial	Intravenous infusion of α‐GalCer‐pulsed DCs	Patients with completely resected stage II–IIIA NSCLC	Improved RFS; enhanced NKT cell immune response	α‐GalCer activates NKT cells through CD1d, secretes IFN‐γ, and enhances cytotoxicity and immune regulation.	[413]
Clinical trial (NCT01574222)	Phase I clinical trial	Intratumoral injection of autologous DCs modified with CCL21 gene (Ad–CCL21–DC)	Patients with advanced (stage IIIB/IV) NSCLC	25% of patients had stable disease; 54% of patients had increased CD8+ T cell infiltration in their tumors	CCL21 recruits T cells and DCs to form a lymphoid microenvironment and enhance antitumor immunity.	[37]
Clinical trial (ChiCTR‐TRC‐12002369)	Phase II clinical trial	Autologous DCs and CIK cells combined with thoracic radiotherapy	Patients with locally advanced or metastatic NSCLC	Median PFS: 330 days (treatment group) vs. 233 days (control group) ORR: 47.6% (treatment group) vs. 24.6% (control group)	Radiotherapy releases tumor antigens, DCs present antigens and activate CIK, enhancing antitumor immunity	[414]
Clinical trial (NR)	NR	DC vaccine loaded with tumor antigens combined with CIK cell infusion	Patients with metastatic NSCLC	Treatment failed to reverse the Th2‐dominant state in advanced patients; it enhanced Th1 responses and cytotoxic T cells in tumor‐free patients.	The tumor microenvironment (such as high VEGF) may lead to Th2 polarization and weaken the effect of immunotherapy.	[415]
Clinical trial (NR)	Phase III clinical trial	Adoptive transfer of autologous activated killer T cells and DCs (AKT–DC)	Patients with postoperative NSCLC	The 5‐year OS rate was 81.4% in the immunotherapy group vs. 48.3% in the control group (HR 0.229, *p* = 0.0013); the 5‐year RFS rate was 56.8 vs. 26.2% (HR 0.423, *p* = 0.0027).	Targeted elimination of chemotherapy‐resistant micrometastases and enhanced antitumor immune responses	[416]
Clinical trial (NR)	Phase I clinical trial	Autologous tumor lysate‐loaded, poly‐ICLC/TNF‐α/IFN‐α matured DC vaccine	Patients with advanced solid tumors (including prostate cancer, mesothelioma, head and neck cancer, etc.)	The combined therapy was safe. Nine patients experienced stable disease (five in the radiotherapy group) and one had a mixed reaction. Signs of immune activation were observed.	Simulating intratumoral viral infection (poly‐ICLC) combined with radiotherapy induces immunogenic cell death and systemic immunity.	[417]
Clinical trial (NCT02993315)	Phase III clinical trial	Autologous CD1c+ conventional and plasmacytoid DCs loaded with tumor antigens	Melanoma patients	Execution and production of an autologous natural DC product is feasible and treatment was well tolerated.	Induction of a functional T cell response against these cancer testis and tumor‐associated antigens	[418]
Clinical trial (NCT02718391)	Phase II clinical trial	Autologous tumor lysate‐pulsed DC vaccine combined with low‐dose IL‐2	Patients with resected stage III/IV melanoma	The treatment group showed a trend toward better RFS (especially in women and younger patients), had a good safety profile, and induced immune regulation.	Activation of antitumor immunity by DC vaccines alters systemic and intratumoral immune cell dynamics.	[419]
Clinical trial (NCT02301611)	Phase I/IIa clinical trial	TLPLDC (tumor lysate particles loaded onto DCs) versus TLPO (tumor lysate particle vaccine alone)	Patients with resected stage III/IV melanoma	There was no significant difference in 36‐month DFS (60.8 vs. 58.7%) and OS (94.6 vs. 93.8%) between TLPO and TLPLDC, and the safety profile was good.	Delivery of autologous tumor lysates to DCs via YCWP particles in vivo or in vitro to induce tumor‐specific immune responses.	[420]
Clinical trial (NCT02301611)	Phase 2B clinical trial	TLPLDC vaccine (loaded with DC in vitro), TLPO vaccine (yeast particles loaded with tumor lysate, taken up by DC in vivo)	Patients with resected stage III/IV melanoma	TLPO and TLPLDC (without G‐CSF) significantly improved 36‐month disease‐free survival and OS.	Yeast particles deliver tumor antigens to induce DC maturation and activate tumor‐specific T cell responses.	[421]
Clinical trial (ACTRN12612001101875)	Phase I / II clinical trial	Autologous monocyte‐derived DCs loaded with long NY‐ESO‐1 peptide, with or without α‐GalCer	Patients with high‐risk, completely resected stage II–IV cutaneous melanoma	Most patients developed NY‐ESO‐1‐specific T cell responses, but α‐GalCer did not significantly enhance T cell or NK T cell responses.	α‐GalCer, as a NKT cell agonist, can theoretically enhance T cell responses, but no significant auxiliary effect has been shown in clinical practice.	[422]
Clinical trial (NCT02574377)	Phase II clinical trial	Autologous CD1c+ myeloid DC (cDC2) and pDC, used alone or in combination, loaded with peptide antigens	Patients with completely resected stage III melanoma	Antigen‐specific CD8+ T cells were detected in 80% of patients in skin tests, indicating that the vaccine is safe and can induce immune responses.	cDC2 and pDC mature through TLR agonists (pR), secrete IL‐12 and IFNα, respectively, and synergistically enhance T cell immune responses.	[423]
Clinical trial (NCT03747744)	Phase I clinical trial	Intratumoral injection of CD1c⁺ and CD141⁺ myeloid DCs combined with T‐VEC	Patients with advanced melanoma refractory to immune checkpoint blockade	Safe and feasible; two patients achieved sustained complete remission; local and systemic antitumor responses were observed.	T‐VEC induces immunogenic cell death, myDCs capture and cross‐present antigens, and activate T cell responses.	[424]
Clinical trial (NR)	Phase IIB clinical trial	TLPLDC vaccine (tumor lysate + yeast cell wall particles loaded DCs)	Patients with resected stage III/IV melanoma	The DFS and OS were significantly improved in the TLPLDC vaccine group without G‐CSF prestimulation.	G‐CSF mobilization leads to immature and tolerogenic DC phenotypes, reducing vaccine immunogenicity.	[425]
Clinical trial (JRC10030190195)	Phase I clinical trial	WT1 peptide‐pulsed DC vaccine combined with albumin‐paclitaxel + gemcitabine chemotherapy	Patients with unresectable advanced pancreatic ductal adenocarcinoma	Safe and feasible; eight patients underwent conversion surgery; long‐term WT1–DTH‐positive patients had significantly prolonged survival.	Activates WT1‐specific CD4+/CD8+ T cells, modulates the tumor microenvironment, and reduces Treg cells and MDSCs.	[426]
Clinical trial (NL7432)	Phase I clinical trial	Allogeneic tumor lysate‐pulsed autologous monocyte‐derived DC vaccine (MesoPher)	Patients with pancreatic ductal adenocarcinoma after surgical resection	Safe and feasible; induces tumor‐reactive T cells; seven out of 10 patients remained relapse‐free at a median follow‐up of 25 months.	Shared tumor antigens activate specific CD4+/CD8+ T cells and trigger antitumor immune responses.	[427]
Clinical trial (NR)	Phase Ib clinical trial	Autologous DCs loaded with personalized neoantigen peptides (PEP–DC)	Patients with resected pancreatic ductal adenocarcinoma	Successfully identified new antigens and prepared vaccines in three patients; pre‐existing CD4+ T cell responses	Neoantigen vaccines enhance tumor immune recognition and reverse immunosuppression in combination with PD‐1 inhibitors and aspirin.	[428]
Clinical trial (UMIN000027179)	Phase II clinical trial	Autologous DC vaccine loaded with WT1 peptide (TLP0‐001) combined with S‐1 chemotherapy	Patients with advanced pancreatic cancer refractory to standard chemotherapy	The trial is ongoing, and final OS data are pending. Previous studies suggest that WT1‐specific CTLs can be induced.	DCs present WT1 antigens to induce specific CTLs to kill tumor cells.	[429]
Clinical trial (NCT01781520)	Phase I/II clinical trial	DCs and CIK cells combined with S‐1 chemotherapy	Patients with advanced pancreatic cancer	The median OS and PFS of the DC–CIK combined with S‐1 group were significantly prolonged (212 and 136 days); changes in peripheral immune cell subsets.	DC–CIK enhances antitumor immunity (increases CD3+/CD4+ T cells and reduces Treg cell), and S‐1 synergistically inhibits tumors.	[430]
Clinical trial (NCT01410968)	Phase I clinical trial	Peptide‐pulsed autologous DC vaccine combined with poly‐ICLC (hTERT, CEA, survivin peptide)	Patients with HLA‐A2+ advanced pancreatic cancer (metastatic or locally advanced unresectable)	The treatment was well tolerated and induced antigen‐specific T cells, with a median OS of 7.7 months and 4 patients experiencing stable disease.	TLR3 agonists enhance DC activation and induce tumor‐specific T cell responses.	[431]
Clinical trial (NCT00868595)	Phase I clinical trial	Antigen‐targeted autologous DC‐based vaccine	Advanced prostate cancer patients	DCs vaccine demonstrated safety, immune response, and antitumor activity in both chemotherapy‐naïve and postchemotherapy advanced prostate cancer patients.	It may be related to cellular immune responses or antibody responses.	[432]
Clinical trial (NCT02423928)	Phase I clinical trial	Cryoablation combined with intratumoral injection of autologous iDCs, with or without immune checkpoint inhibitors	Patients with mCRPC	The treatment was safe and tolerable, with 33% of patients achieving lasting clinical benefit and a median OS of 40.7 months.	Cryoablation releases tumor antigens, iDCs capture and present antigens, activate T cells, and combined with checkpoint blockade to enhance immunity.	[433]
Clinical trial (NCT01197625)	Phase I/II clinical trial	DC‐adjuvanted vaccines transfected with autologous tumor mRNA or hTERT/Survivin mRNA	Patients after radical prostatectomy for high‐risk prostate cancer	55% of patients remained biochemically relapse‐free (median 96 months), all relapsed patients had stable disease, and the vaccine was safely tolerated.	Induces CD4+ T cell responses against multiple tumor antigens (such as hTERT, Survivin, STEAP1, etc.)	[434]
Clinical trial (NCT02692976)	Phase IIa clinical trial	Blood‐derived CD1c+ mDC and/or pDC vaccine loaded with NY‐ESO‐1, MAGE‐C2, and MUCI antigens, matured by protamine/mRNA	Patients with castration‐resistant prostate cancer (CRPC) who had not received chemotherapy	Induction of functional antigen‐specific T cells was associated with longer rPFS (18.8 vs. 5.1 months, *p* = 0.02)	DC vaccines activate antigen‐specific T cells and enhance antitumor immune responses.	[435]
Clinical trial (NCT01446731)	Phase II clinical trial	Autologous DC vaccine loaded with PSA, PAP, survivin, and hTERT mRNA, combined with docetaxel	Patients with mCRPC	Combination therapy was safe but did not significantly improve PFS or DSS; approximately 50% of patients developed an antigen‐specific immune response.	Docetaxel may reduce MDSCs levels, modulate the tumor immune microenvironment, and enhance vaccine immunogenicity.	[436]
Clinical trial (NCT00852007)	Phase I/II clinical trial	Tn‐MUC1 glycopeptide‐loaded autologous DC vaccine	Non‐mCRPC patients	Safe, induced T cell responses, significantly increased PSAT1D doubling time	Overcoming tolerance to MUC1, targeting TAA (Tn antigen)	[437]
Clinical trial (EudraCT 2009‐017295‐24)	Phase I/II clinical trial	DCVAC/PCa (DC vaccine)	mCRPC patients	Median OS extended to 19 months in patients	Chemo reduces Treg cells, DC vaccine induces PSA‐specific T cells.	[438]
Clinical trial (NTR5542)	Phase I clinical trial	TolDCs pulsed with proinsulin peptide	T1DM patients	Safe and feasible, with no immune suppression, no insulin allergy, and no accelerated loss of β‐cell function.	TolDCs induce antigen‐specific tolerance via proinsulin peptide.	[297]
Clinical trial (2013‐005476‐18)	Phase I clinical trial	TolDCs pulsed with islet antigen	T1DM patients	Autoimmune response to the vaccine peptide was significantly reduced, and the T cell response to other islet autoantigens was temporarily decreased.	tolDCs modulate islet cell autoimmunity by targeting CD4+ T cell responses, while prime‐boost injections stimulate T cells and reduce CCR6+ effector CD4+ T cells, without affecting other peripheral blood cells.	[23]
Clinical trial (NCT00510497)	Phase I/II clinical trial	HIV antigen‐loaded DC vaccination	HIV‐1‐infected patients	The therapeutic HIV‐1 vaccine based on DCs loaded with apoptotic bodies was safe and induced T cell activation and cytolysis, including of HIV‐1 infected cells, in a subset of study participants.	Therapeutic efficacy depends on DC maturity and function, with factors like freeze–thaw cycles and immune responses to CTL escape mutations potentially affecting vaccine effectiveness, especially in chronic HIV‐1 patients on antiretroviral therapy.	[439]
Clinical trial (NCT02767193)	Phase 2A clinical trial	DCV3 (autologous DC‐HIV vaccine)	Chronic HIV‐1 infected patients	Vaccine safe but did not control viral rebound	DC vaccine may stimulate T cells but limited effect.	[440]
Clinical trial (NCT00796146)	NR	pDC	Acute HIV‐infected patients	pDC frequency ↑, function ↓ before rebound	pDCs sense HIV replication and become refractory.	[441]
Clinical trial (NCT00796770)	Phase I clinical trial	IFNα‐DCs loaded with LIPO‐5	HIV‐1‐infected patients	Vaccine induced CD4+ T (IFNγ, IL‐2, IL‐13) and CD8+ T (IFNγ, perforin, granzyme A/B); broadened responses; reduced viral rebound post‐ATI.	CD4+ T IL‐2/IL‐13 responses inversely correlated with viral load; mechanism via helper T cell‐mediated immune control of HIV replication.	[442]
Clinical trial (NCT00796770)	Phase I/II clinical trial	IFNα‐DCs loaded with HIV lipopeptides	HIV‐Infected Patients	Enhanced HIV‐specific T cell responses, viral suppression post‐ATI	Excessive TLR4‐mediated inflammation correlates with higher viral rebound post‐ATI.	[443]
Clinical trial (NR)	Phase I clinical trial	AGS‐004: autologous DCs electroporated with HIV RNA	Acute HIV‐infected patients	Enhanced HIV‐specific CD8+ memory T cell responses, delayed viral rebound	Increased multifunctional CD28+/CD45RA− memory T cells correlated with delayed viral rebound.	[444]
Clinical trial (NCT00833781)	Phase I clinical trial	Autologous DCs transfected with HIV‐1 Gag and Nef mRNA	HIV‐1‐infected adults on antiretroviral therapy	Enhanced T cell proliferation (CD4/CD8) postvaccination	Induced CD4+/CD8+ T cell proliferation, transient responses	[445]
Clinical trials (NCT01845350)	Phase I/II clinical trial	IFN‐DC vaccine	Chronic HCV infection patients	IFN‐DC therapy was safe and elicited HCV‐specific T cell responses that, although insufficient to eliminate the virus, may have contributed to restricting replication.	The therapy can induce core protein/NS3‐specific proliferation, IFN‐γ production, and CD8+ T lymphocyte degranulation responses.	[446]
Clinical trial (NR)	Phase I clinical trial	HSP70 mRNA‐transfected DCs	HCV‐related HCC patients	Two patients had complete response, five stable disease.	CD8+ T cell and granzyme B activation, inducing antitumor response	[447]
Clinical Trial (NCT05007496)	Phase I and II clinical trials	Autologous DC vaccine loaded with SARS‐CoV‐2 S‐protein	Healthy adult subjects	Vaccine safe with no serious adverse event over 1 year; 95.56% efficacy against COVID‐19 hospitalization	DCs as APCs induce T cell immunity and SARS‐CoV‐2‐specific memory T cells.	[448]
Clinical Trial (NCT01352858)	Phase I clinical trial	Autologous toIDC loaded with autologous synovial fluid	Patients with rheumatoid and inflammatory arthritis	Intra‐articular injection safe and feasible; knee symptoms stabilized in two high‐dose patients	toIDC produce IL‐10, induce T cell tolerance, and suppress inflammation.	[449]
Clinical Trial (NCT02283671)	Phase Ib clinical trial	IV infused peptide‐loaded toIDCs (myelin or AQP4 peptides)	Patients with MS and NMOpr	Therapy safe with no serious adverse event; increased IL‐10 and Tr1 cell frequency	toIDCs induce antigen‐specific Tr1 cells and IL‐10‐mediated immune tolerance	[450]
Clinical Trial (NCT02847598)	Phase II clinical trial	Subcutaneous anti‐BDCA2 mAb litifilimab	Patients with SLE	Litifilimab reduced joint counts more than placebo; acceptable safety	Litifilimab binds BDCA2 on pDCs to suppress type I IFN production	[451]
Clinical trial (NCT02106897)	Phase I clinical trial	Anti‐BDCA2 mAb (BIIB059)	SLE patients with skin lesions	Single dose reduced skin MxA and CLASI‐A score	BIIB059 binds BDCA2, internalizes it, inhibits IFN‐I and cytokine production.	[452]
Preclinical study/clinical trial (NR)	In vivo animal experiments/phase II clinical trial	Cord blood‐derived DCs plus CIK cells therapy	Advanced gastric cancer patients	CB–DC–CIK therapy improved disease‐free survival, with trends of improvement in OS, objective response rate, and disease control rate. It also increased T cell subsets and serum levels of IFN‐γ, TNF‐α, and IL‐2, with no serious side effects observed.	It enhances cellular immunity by increasing effector immune cells and immunostimulatory cytokines, while CIK cells directly kill tumor cells and may synergize with chemotherapy to reduce tumor burden and boost immunotherapy effectiveness.	[453]
Preclinical study	In vitro cell experiments	DC–CIK cells	Glioblastoma patients	DC–CIK cells exhibited significant cytotoxic effects against glioblastoma cell lines, particularly those with a stem‐like cell phenotype. Furthermore, increased specific lysis of these cells following DC–CIK coculture was confirmed by confocal fluorescence microscopy.	DC–CIK cells are effective against glioblastoma cells primarily through apoptosis mediated in part by elevated IFN‐γ levels.	[454]
Preclinical study	In vitro cell experiment	DEXA + RGZ‐induced tolDCs	SLE patients and healthy donor monocytes	tolDCs showed stable immunosuppressive phenotype, reduced costimulatory molecules and inflammatory cytokines, suppressed T cell proliferation.	DEXA and RGZ induce tolDCs that promote T cell tolerance via low costimulation and cytokine secretion.	[455]
Preclinical study	In vivo animal experiment	CD40–siRNA transfected OVA‐pulsed DCs	Mice with allergic rhinitis	Intranasal administration significantly reduced sneezing and nasal rubbing	CD40–siRNA‐treated DCs upregulated Treg cells in lymph nodes	[456]
Preclinical study	In vivo animal experiment	Der p1‐modified DCs	Mice with allergic rhinitis	DC therapy significantly reduced allergic symptoms, decreased IgE, and IgG1 levels.	Downregulation of IL‐4 and IL‐13, upregulation of IL‐10, inducing immune tolerance	[457]
Preclinical study	In vivo animal experiment	Der p1‐pulsed tolDCs	Mice with allergic rhinitis	DC therapy significantly reduced allergic symptoms and inflammatory cell infiltration.	Induction of Treg cells via TGF‐β/IL‐10 signaling, suppressing inflammation	[458]
Preclinical study	In vivo animal experiment	Ad–DLL1 transduced DCs	OVA‐sensitized asthmatic mice	DLL1–DCs transfer alleviated AHR and airway inflammation.	DLL1–DCs promote Th1 differentiation and IFN‐γ secretion, correcting Th1/Th2 imbalance.	[459]
Preclinical study	In vitro cell experiment	MI mice	IL‐37 and Troponin I (TnI) treated DCs	IL‐37 + TnI‐treated DCs increased Treg cells, reduced myocardial inflammation, alleviated myocardial fibrosis, and improved cardiac function.	IL‐37 modulates the immune system by restoring a tolerogenic immune response, alleviating postmyocardial infarction remodeling.	[460]
Preclinical study	In vivo animal experiment	GM‐CSF‐induced tolerogenic BMDCs transfer	MPTP‐induced PD mice	BMDCs reduce neuroinflammation and protect dopaminergic neurons.	BMDCs induce Treg cells and suppress microglial activation.	[377]
Preclinical study	In vivo animal experiment	Aβ₁₋_42_‐stimulated BMDCs + young stem cells	APPswe/PSEN1dE9 transgenic mice	Combined treatment reduced Aβ plaques and improved cognitive function.	Enhanced microglial phagocytosis via increased CD68 expression	[461]
Preclinical study	In vivo and in vitro	DART vaccine (tFNA–antigen peptide–miR‐23b‐aptamer)	MRL/lpr SLE mice and DC2.4 cell line	DART induced tolDCs and ameliorated SLE symptoms.	miR‐23b inhibits NF‐κB pathway to induce immune tolerance.	[372]
Preclinical study	In vivo animal experiment	IL‐10‐treated iDCs + CTLA4‐Ig	B6.MRL‐Faslpr/J SLE mice	Combined treatment reduced autoantibodies and inflammation.	iDCs induce Treg cell differentiation; CTLA4‐Ig blocks costimulation.	[462]
Preclinical study	In vivo animal experiment	Combined use of IFN‐beta and VitD3–tolDC	C57BL/6 mouse EAE model	Compared with single treatment, VitD3–tolDC combined with IFN‐beta treatment significantly alleviated the clinical symptoms of EAE in mice.	VitD3–tolDC–MOG combined with IFN‐beta treatment can reduce specific T cell responses, increase IL‐10 secretion, and alleviate clinical symptoms.	[463]
Preclinical study	In vivo animal experiment	IL‐10‐DC10s + polyethylene glycol hydrogel localized delivery	EAE mice (C57BL/6)	DC10s in neck hydrogel attenuate EAE paralysis, not in flank.	DC10s secrete IL‐10 and suppress T/B cell proliferation via PirB.	[464]
Preclinical study	In vivo animal experiment	SOCS1–siRNA‐transfected DCs	Mice with systemic C. albicans infection	SOCS1‐silenced DCs increase survival and reduce fungal load	SOCS1 silencing enhances Th1 response via IFN‐γ/JAK–STAT pathway	[465]
Preclinical study	In vivo animal experiment	DC‐targeting nanobody delivered antigen	Chickens/H9N2 AIV challenge model	Enhanced mucosal/humoral immunity, reduced virus shedding, protection	Enhanced antigen presentation via DC‐targeting, CpG activates TLR21, promotes Th1 response	[466]
Preclinical study	In vivo animal experiments	α‐GalCer‐pulsed DCs	Dilated cardiomyopathy heart failure mice	α‐GalCer‐pulsed DCs activated iNKT cells, prolonged the survival of mice, prevented the decline in left ventricular ejection fraction over 4 weeks, and inhibited interstitial fibrosis.	By inhibiting TGF‐β signaling and fibrotic gene expression, and upregulating Angpt1, cardiac vascular function was restored in mice, while IFNγ suppressed TGF‐β‐induced Smad2/3 signaling and upregulated Angpt1 expression in cardiomyocytes.	[467]
Preclinical study	In vivo animal experiments	An intranasal stringent response vaccine targeting DCs	Chronic tuberculosis mice	Immunization with a DNA vaccine expressing MIP‐3α/rel_Mtb_ generates strong, additive Th1 and Th17 responses and significantly potentiates the mycobactericidal activity of the first‐line drug, isoniazid.	The aggregate percentage of Rel _Mtb_‐specific CD4+ and CD8+ T cells producing IL‐17A, TNF‐α, IFN‐γ, or IL‐2 in the spleens and lungs of _Mtb_‐infected animals was greater in the recipients of the intranasally administered fusion vaccine.	[468]
Preclinical study	In vitro cell and in vivo animal experiments	tolDCs	Acute kidney injury mice	tolDCs can be leveraged to protect against acute kidney injury and limit kidney parenchymal damage after ischemia–reperfusion injury by restricting renal tubular epithelial cell death.	tolDCs induce an anti‐inflammatory phenotype, inhibit T cell activation, protect renal tubular cells, and alleviate kidney damage. They migrate to the injured kidney via CCR7, secrete TGF‐β/IDO1, and promote fatty acid oxidation, lipid metabolism, energy homeostasis, and apoptosis inhibition, while remodeling the immune microenvironment.	[469]
Preclinical study	In vitro cell and in vivo animal experiments	Engineered DCs using CRISPR/Cas9 for targeted gene disruption of the VPS37A and VPS37B genes	Allogeneic naïve CD4 T cells from Allergic rhinitis patients/allergic rhinitis mice	In vitro: coculture with VPS37A/B knockout DCs reduced Th2 cytokine production by T cells from AR patients. In vivo: intranasal administration of VPS37A/B knockout BMDCs reduced allergic symptoms in an AR mouse model.	VPS37A/B promotes the uptake and presentation of Der p 1 by DCs through mannose receptor endocytosis. Knockout of VPS37A/B disrupts this process, impairing DCs' ability to present Der p 1 and suppressing Th2 cytokine production.	[470]
Preclinical study	In vivo animal experiments	TolDCs	Rheumatoid arthritis mice	Alleviated joint inflammation, synovitis, and bone erosion by reducing the production of anti‐type II collagen antibodies, inhibiting pro‐inflammatory factors (TNF‐α, IL‐1β), and increasing anti‐inflammatory factors (IL‐4).	It reduces autoantibody production by lowering serum levels of anti‐type II collagen antibodies, inhibits proinflammatory factors by reducing TNF‐α and IL‐1β, and increases anti‐inflammatory factors by raising serum levels of IL‐4.	[471]
Preclinical study	In vivo animal experiments	Alidomide combined with tumor‐carrying tumor antigen DCs	Colon cancer mice	The tumor antigen‐loaded DCs plus lenalidomide combination treatment exhibited a significant inhibition of tumor growth.	These effects were linked to a decrease in immune suppressor cells and an increase in immune effector cells, including NK cells, CD4+ T cells, CD8+ T cells, and the activation of CTLs and NK cells.	[472]

*Abbreviations*: AFP, alpha‐fetoprotein; AIV, avian influenza virus; APC, antigen‐presenting cell; ATI, analytical treatment interruption; Aβ, Amyloid‐beta; BMDCs, bone marrow‐derived dendritic cells; cART, combined antiretroviral therapy; CEA, carcinoembryonic antigen; CIK, cytokine‐induced killer; CTLs, cytotoxic T lymphocytes; DC, dendritic cell; DFS, disease‐free survival; DSS, disease‐specific survival; DTH, delayed‐type hypersensitivity; EAE, experimental autoimmune encephalomyelitis; HCC, hepatocellular carcinoma; HCV, hepatitis C virus; HIV, human immunodeficiency virus; hTERT, human telomerase reverse transcriptase; iDCs, immature dendritic cells; IDO1, indoleamine 2,3‐dioxygenase 1; IFN, interferon; JAK–STAT, Janus kinase‐signal transducer and activator of transcription; mAb, monoclonal antibody; mCRPC, metastatic castration‐resistant prostate cancer; MDSC, myeloid‐derived suppressor cell; MS, multiple sclerosis; NKT, natural killer T; NSCLC, non‐small cell lung cancer; ORR, objective response rate; OS, overall survival; PAP, prostatic acid phosphatase; PD, Parkinson's disease; pDC, plasmacytoid dendritic cell; PFS, progression‐free survival; PSA, prostate‐specific antigen; RFS, relapse‐free survival; SCLC, small cell lung cancer; SLE, systemic lupus erythematosus; T1DM, type 1 diabetes mellitus; TAA, tumor‐associated antigen; TACE, transarterial chemoembolization; Th, T helper; toIDC, tolerogenic dendritic cell; Treg cell, regulatory T cell; VEGF, vascular endothelial growth factor; α‐GalCer, alpha‐galactosylceramide.

In summary, DCs play a central immunomodulatory role in various diseases, making DC‐targeting therapeutic strategies promising for clinical applications. Currently, DC‐based vaccines, tolDC induction, and combination strategies, such as integration with conventional chemotherapy, immune checkpoint inhibitors, novel adjuvants, or nanocarrier technologies, are rapidly advancing. Extensive preclinical and clinical studies have not only demonstrated the safety and preliminary efficacy of DC‐based therapies in specific disease contexts but have also laid a solid foundation for their translation from basic research to clinical practice. Although challenges such as immunosuppressive TMEs, antigen selection, and delivery efficiency remain, continued progress in personalized vaccine design and precision immunomodulatory technologies underscores the broad clinical potential of DC‐based treatment strategies.

## 6 Conclusions and Prospects

As central regulators of immune responses, DCs play indispensable roles in maintaining immune homeostasis, defending against pathogens, and mediating disease processes by initiating T‐cell responses and coordinating the crosstalk between innate and adaptive immunity. This review systematically integrates the multilineage origins of DCs from HSCs (including myeloid/LP cells) and their hierarchical developmental network orchestrated by cytokines such as Flt3L, GM‐CSF, and M‐CSF, and transcription factors such as IRF8, BATF3, and ID2. It further elaborates on the major DC subsets and their heterogeneity: cDC1s (specialized in cross‐presentation), cDC2s (activating CD4⁺ T cells), pDCs (secreting IFN‐I), moDCs (mediating inflammatory responses), and LCs (immune surveillance at epidermal barriers). The biological functions of DCs, including pathogen recognition and antigen capture, CCR7‐mediated migration to lymphoid tissues, antigen presentation via the MHC class I and II pathways, the initiation of T‐cell‐ and B‐cell‐mediated adaptive immunity, the induction of central and peripheral tolerance, and the regulation of innate immune cells such as NK and NKT cells, are also discussed.

Given their multifaceted biological functions, DCs play critical roles in various diseases. In cancer, DCs serve as central drivers of antitumor immunity by cross‐presenting tumor antigens, secreting cytokines such as IL‐12, and recruiting and activating CD8⁺ T cells and NK cells. In T1DM, DCs infiltrating pancreatic islets present β‐cell autoantigens, activate autoreactive Th1/Th17 cells, and recruit cytotoxic T cells, collectively contributing to the loss of insulin secretion. The pathogenesis of T2DM is closely linked to chronic low‐grade inflammation driven by DCs, which disrupts insulin signaling and promotes β‐cell dysfunction across adipose tissue, liver, and skeletal muscle, exacerbating systemic metabolic dysregulation. In infectious diseases, DCs precisely recognize PAMPs via PRRs to initiate anti‐infective immune responses. In transplantation, under steady‐state conditions, DCs help maintain graft tolerance by inducing Treg cell differentiation through mechanisms involving PD‐L1 and TGF‐β. In high‐inflammatory environments dominated by IFN‐γ, they can adopt a proinflammatory phenotype that drives rejection. DCs also play important roles in a spectrum of other conditions, including autoimmune diseases, neurodegenerative disorders, cardiovascular diseases, allergic diseases, and dermatological disorders. Innovative therapeutic strategies targeting DCs are rapidly evolving. DC‐based vaccines have become a central intervention in cancer treatment, where DC vaccines loaded with peptides (exogenous antigen sensitization) or genetically engineered vaccines (enhancing endogenous antigen expression and function) activate T cells to produce IFN‐γ and recruit NK/B cells, thereby inducing specific antitumor immune responses. In chronic infections, ex vivo peptide‐loaded DC vaccines or in vivo targeted NP vaccines can reconstruct pathogen‐specific T‐cell responses. With respect to autoimmune diseases, tolDC vaccines, which use modulators such as vitamin D_3_, rapamycin, or gene editing to overexpress IL‐10/PD‐L1, successfully induce antigen‐specific Treg cells, demonstrating the potential for stabilizing blood glucose levels and inhibiting autoimmunity in clinical trials for T1DM and RA. Furthermore, DC vaccines are often combined with multiple strategies to increase efficacy: inducing tolerogenic phenotypes in combination with immunomodulators (IL‐10, rapamycin, and vitamin D_3_) to control excessive immune responses or using TLR agonists (CpG/poly‐ICLC) or immune checkpoint inhibitors (anti‐PD‐1/CTLA‐4) to overcome immune tolerance and reverse T‐cell exhaustion. In cancer, DC vaccines can synergize with traditional therapies such as chemotherapy or low‐dose methylation agents to reshape the TME, whereas in infectious diseases, combining DC vaccines with antiviral drugs and neutralizing antibodies helps restore pathogen‐specific T‐cell responses. Additionally, the integration of nanotechnology has increased the precision of DC regulation, with functionalized NPs significantly improving the efficiency of antigen delivery and codelivery of adjuvants, thereby increasing the effectiveness of DC vaccines. Emerging cellular engineering strategies are further expanding therapeutic possibilities. iPSC‐derived tolDCs have been shown to effectively suppress autoimmunity in diabetes models.

Despite these promising advancements, several challenges remain in DC research. (1) Antigen selection is limited, and a risk of immune escape exists. Current DC vaccine antigen designs (e.g., tumor neoantigens and T1DM autoantigens) inherently suffer from incomplete coverage and fail to address the high heterogeneity of diseases (e.g., tumor clonal evolution and diabetes‐related multiepitope autoimmunity), which may lead to local immune escape. (2) The durability of tolerance induction is a key challenge. Treg cell expansion induced by tolDCs in transplantation and autoimmune diseases is often transient and susceptible to reversal by environmental triggers (e.g., viral infections and chronic inflammation). The long‐term maintenance of immune tolerance mechanisms remains unresolved. (3) Limited targeting efficiency, off‐target risks, and challenges in crossing biological barriers (e.g., blood‒brain/blood‒pancreas barriers) restrict the application of DC therapies in patients with neurodegenerative and metabolic diseases. (4) The impact of individual heterogeneity on the therapeutic response needs to be quantified. (5) The high production cost and difficulties in standardizing autologous DC therapies remain obstacles. (6) The long‐term safety profile of gene‐edited DCs requires thorough investigation. Potential unintended immunological consequences, such as the off‐target effects of gene editing, insertional mutagenesis, sustained or excessive immune suppression leading to secondary infections, and the theoretical risk of malignant transformation, must be carefully evaluated in both preclinical and clinical settings. Future research will need to integrate innovative strategies and overcome these challenges to bridge the gap between modifiable DC therapies and clinical cures.

In conclusion, DCs, which are central regulators of the immune system, are indispensable for mediating pathological processes. Continued in‐depth research on their origin, development, classification, and biological functions will deepen our understanding of the roles of DCs in both health and disease and promote the development of innovative DC‐targeted therapies.

## Author Contributions

Fangfang Jin and Wei Liu drafted the manuscript, table, and figures. Lingxiang Xie, Hongqi Zhang, and Xiang Fan helped table and figure modification. Yang Xiao, Jiaxing Tian, and Xinrong Fan revised the manuscript. All authors have read and approved the final manuscript.

## Ethics Statement

The authors have nothing to report.

## Conflicts of Interest

The authors declare no conflicts of interest.

## Data Availability

The authors have nothing to report.
